# Post-Synthetic Shaping of Porosity and Crystal Structure of Ln-Bipy-MOFs by Thermal Treatment

**DOI:** 10.3390/molecules200712125

**Published:** 2015-07-03

**Authors:** Philipp R. Matthes, Fabian Schönfeld, Sven H. Zottnick, Klaus Müller-Buschbaum

**Affiliations:** Institute of Inorganic Chemistry, University of Würzburg, Am Hubland, 97074 Würzburg, Germany; E-Mails: philipp_matthes@web.de (P.R.M.); fabian_schoenf@hotmail.com (F.S.); sven.zottnick@uni-wuerzburg.de (S.H.Z.)

**Keywords:** metal-organic frameworks, shaping of porosity, thermal treatment, Ln-MOFs, luminescence, crystal structure

## Abstract

The reaction of anhydrous lanthanide chlorides together with 4,4′-bipyridine yields the MOFs${\;}_{\infty }^{2}$[Ln_2_Cl_6_(bipy)_3_]·2bipy, with Ln = Pr − Yb, bipy = 4,4′-bipyridine, and${\;}_{\infty }^{3}$[La_2_Cl_6_(bipy)_5_]·4bipy. Post-synthetic thermal treatment in combination with different vacuum conditions was successfully used to shape the porosity of the MOFs. In addition to the MOFs microporosity, a tuneable mesoporosity can be implemented depending on the treatment conditions as a surface morphological modification. Furthermore, thermal treatment without vacuum results in several identifiable crystalline high-temperature phases. Instead of collapse of the frameworks upon heating, further aggregation under release of bipy is observed.${\;}_{\infty }^{3}$[LaCl_3_(bipy)] and${\;}_{\infty }^{2}$[Ln_3_Cl_9_(bipy)_3_], with Ln = La, Pr, Sm, and${\;}_{\infty }^{1}$[Ho_2_Cl_6_(bipy)_2_] were identified and characterized, which can also exhibit luminescence. Besides being released upon heating, the linker 4,4′-bipyridine can undergo activation of C-C bonding in ortho-position leading to the *in-situ* formation of 4,4′:2′,2′′:4′′,4′′′-quaterpyridine (qtpy). qtpy can thereby function as linker itself, as shown for the formation of the network${\;}_{\infty }^{2}$[Gd_2_Cl_6_(qtpy)_2_(bipy)_2_]·bipy. Altogether, the manuscript elaborates the influence of thermal treatment beyond the usual activation procedures reported for MOFs.

## 1. Introduction

MOFs (metal-organic frameworks) and coordination polymers [1,2,3,4] are known for their interesting properties such as porosity [5,6], magnetism [7] and luminescence [8,9,10]. They are under investigation for a wide range of potential applications such as gas-storage [11], light-converting materials [12] or sensor-development [13]. As MOFs are typically non-porous in their as-synthesized form, the activation of a MOF is a key factor for MOF materials to unlock their microporosity and sorption properties [14,15]. Therefore, knowledge of the behavior of these compounds at elevated temperature is a vital point, as a thermal activation induces high thermal stress on the compounds. It can have a direct structural influence on the framework, in the worst case causing decomposition, which is typically addressed to as collapse of the MOF. An optimal result is the evaporation of the intercalated solvent molecules, leading to accessible void volume while the framework is structurally unaltered, reported e.g., for MOF-5 [16]. Additional effects like the folding of a network with a direct influence on the volume known as “breathing” can also be observed [17]. An alternative to a structural collapse of a framework upon thermal treatment is structural condensation under release of volatile components (including MOF linkers). This is typically observed for complexes as well as 1D coordination polymers transforming into 3D frameworks and MOFs [18,19,20]. Thus, instead of collapse the formation of new bonds between bridging ligands and their metal-based connection centers can result, leading to structural changes as seen in crystalline MOFs such as SNU-77 [21] or forming amorphous phases such as Zn-HKUST-1 [22]. This can limit the availability of porous structures if a dense structure is thereby available, as it is thermodynamically preferred. For the linker 4,4′-bipyridine, the transformation of the one-dimensional zig-zag chain structure${\;}_{\infty }^{1}$[ZnCl_2_(μ-bipy)] (bipy = 4,4′-bipyridine) to a two-dimensional sheet structure${\;}_{\infty }^{2}$[Zn(μ-Cl)_2_(μ-bipy)] [23,24] shows this possibility.

Besides the ordered ideal crystalline MOF structures, defects and defect engineering of MOFs have recently moved into the focus of MOF research. Disorder phenomena of various nature and scale are well-established for solid-state chemistry, including their influences on material’s properties, but are hardly developed for coordination compounds like MOFs. For the latter, remarkable microscopic to macroscopic effects have recently been shown: defects by missing or altered nodes and fragmented linkers [25,26,27,28,29], the introduction of additional unsaturated and reactive metal centres [30,31], and thereby the accommodation of additional and variable oxidation states of the metals that offer new insights in influencing MOF properties. Modulation and the implementation of hierarchical pore structures up to external surface modifications by morphology control offer modification options into the macroscopic property region [32,33] and drive MOFs beyond their typical properties. The interest in and value of MOF surface chemistry and options to modify the surface have been reviewed recently [34].

In this work, we elaborate the influence of deliberate thermal treatment on the MOF system derived from anhydrous lanthanide halides and 4,4′-bipyridine, as it offers both, options for post-synthetic morphology control by surface modification and shaping of the MOF pores as well as it enables various highly aggregated crystalline high-temperature phases. The knowledge of the thermal behavior thereby explains the activation procedure to the microporous MOFs${\;}_{\infty }^{2}$[Ln_2_Cl_6_(bipy)_3_] [35,36] as well as the inability to activate${\;}_{\infty }^{3}$[La_2_Cl_6_(bipy)_5_]·4(bipy) [37] to a porous MOF.

## 2. Results and Discussion

For MOF materials based on anhydrous LnCl_3_ and 4,4′-bipyridine, two different structure types have been previously identified and characterized as${\;}_{\infty }^{3}$[La_2_Cl_6_(bipy)_5_]·4bipy (**1**) for lanthanum, only [37], and${\;}_{\infty }^{2}$[Ln_2_Cl_6_(bipy)_3_]·2bipy for the series Ln = Pr (**4**), Nd, Sm(**5**)-Yb, which has been partly described before [35,36]. Of both structure types, only the latter series of MOFs were proven to give microporous MOFs upon thermal activation with surface areas observed up to s_BET_ = 660 m^2^·g^−1^ for Gd [36]. However, the activation conditions vary between the different lanthanides by more than 100 °C although all are isotypic, which gave the starting point for a detailed study on the thermal properties of these MOF systems.

The systematic determination of the thermal properties of the MOFs now reveals that different high-temperature stable phases can be observed, depending on the lanthanide ions. For some lanthanides, the transformation into such high-temperature frameworks even prevents a successful activation and leads to the formation of dense phases instead. TGA investigations in temperature regions >200 °C and temperature-dependent X-ray powder diffraction indicate the presence of up to four crystalline phases per lanthanide ion thermally following the initial framework depending on the lanthanide ion and temperature used. Creating suitable single-crystals proved vital for getting access to structure solutions of the high-temperature compounds. As the product formation from such thermal conversions of the initial MOFs is rapid it usually yields microcrystalline powders [38,39]. All high-temperature phases in the system LnCl_3_/4,4′-bipy require formation temperatures >200 °C, inducing thermal stress on the organic part of the compounds. This can cause decomposition processes of the organic ligand. As the formation of new frameworks at elevated temperatures is accompanied by release of bipyridine equivalents, they need to be removed rapidly from the sample to avoid carbonized remnants in the product.

In addition, the influence of a combination of thermal treatment and varying vacuum conditions was investigated on the MOF series${\;}_{\infty }^{2}$[Ln_2_Cl_6_(bipy)_3_]·2bipy with Ln = Pr, Nd, Sm-Yb beyond the previous investigations [35,36]. Vacuum conditions are also typically applied for MOF activation, inducing even higher stress. Our investigations now show that they can lead to a complete surface modification of the MOF material. Temperature and vacuum influence the grade at which molecules incorporated in the pore system try to leave the MOF. If the evaporation rate exceeds the amount of molecules that can be released via the micropores and MOF channels, the morphology is altered. We can show that control of these conditions can be used to change the intrinsic MOF microporosity into a significant mesoporosity without decomposition. Thereby, such deliberate thermal treatment can be used to influence the surface morphology of the post-synthetically modified MOF.

### 2.1. Post-Synthetic Surface Modification and Morphology Control

The thermal evaporation of molecules from the pore system of a MOF is typically accompanied by certain vacuum conditions suitable to help the volatile molecules overcome attractive interactions with the MOF framework. In order to prevent collapse of the framework structures, the activation is typically done with care and caution. We have now followed the question, what happens to a MOF that is exposed to vacuum conditions that provoke an enforced release of the molecules incorporated in the pore system. The release was thereby more and more enforced by variation of the parameters temperature, vacuum and time. For this purpose, representatives of the constitution${\;}_{\infty }^{2}$[Ln_2_Cl_6_(bipy)_3_]·2bipy were used, as the MOF type was known to allow thermal activation to MOFs of the formula${\;}_{\infty }^{2}$[Ln_2_Cl_6_(bipy)_3_] [36].

**Figure 1:**
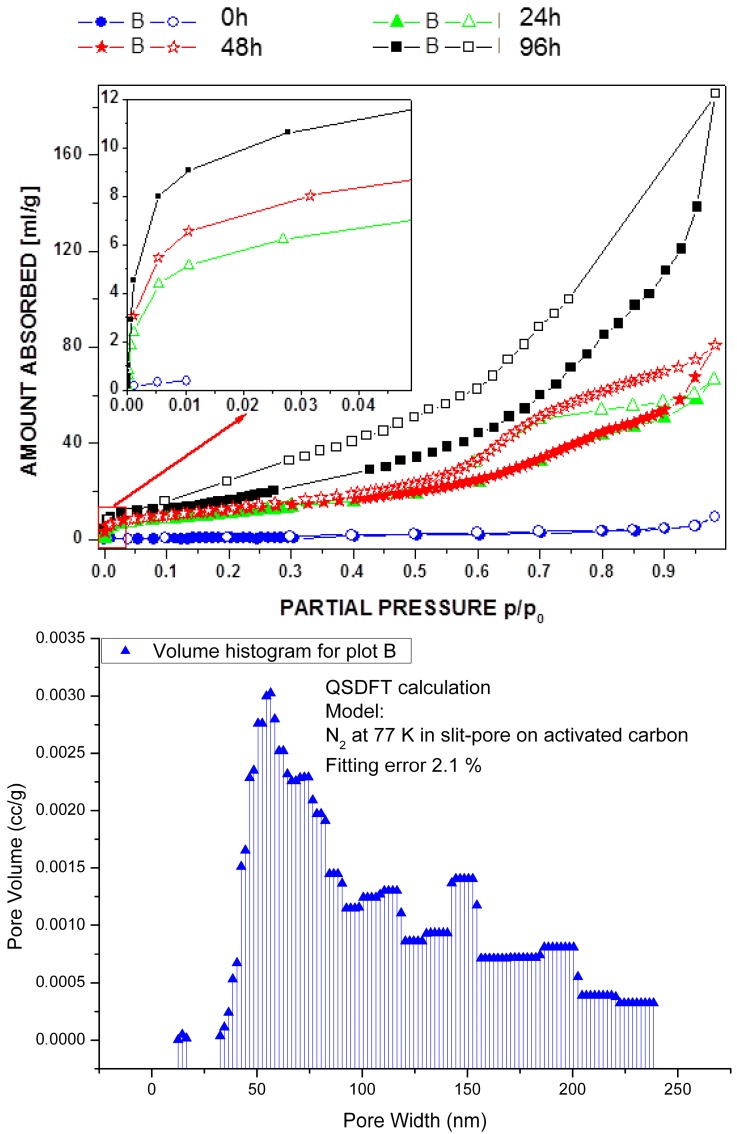
Adsorption isotherms (BET) for the adsorption (closed symbols) and desorption (open symbols) of N_2_ (77 K) on${\;}_{\infty }^{2}$[Sm_2_Cl_6_(bipy)_3_]·2bipy (**5**) for an activation of the MOF at 300 °C and 10^−6^ mbar for activation times between 0–96 h; prolonged activation times result in formation of meso- and macropores indicated by growing hysteresis (**top**); determination of the pore distribution of the thermally treated framework **5** after 96 h using QSDFT calculation based on slit-pore model for activated carbon (**bottom**).

If decomposition conditions are reached by either temperature or a combination of temperature and vacuum, the MOF cannot remain stable, of course. However, we can now show that below such decomposition conditions, the crystalline MOF can be retained while the surface morphology can be vastly changed. The driving force of these surface changes is the evaporation of the incorporated molecules, viz. for${\;}_{\infty }^{2}$[Ln_2_Cl_6_(bipy)_3_]·2bipy two bipyridine molecules are released per formula unit. Whereas typical laboratory vacuum of 10^−2^–10^−3^ mbar can be used to successfully activate the MOFs for Ln = Pr (**4**), Nd, Sm(**5**)-Yb without surface modification, the use of higher vacuum conditions of 10^−5^ to 10^−6^ mbar together with heating have a different effect: Instead of unlocking of the micropores combined to a type-I adsorption isotherm (according to the BET theory) with an uptake of N_2_ gas (77 K) up to 175 cm^3^·g^−1^ [36], type-IV isotherms with an H4-hysteresis are observed. This behaviour was exemplarily investigated for Ln = Sm, Eu and Eu/Tb mixtures and adsorption isotherms were recorded for all of them at different modification conditions (see also Supporting Information, Figures S13 and S14). A lanthanide dependent behaviour was observed. Samarium required the highest surface modification temperature (300 °C at 10^−6^ mbar, Figure 1), whereas europium and a mixture of europium and terbium required only 200 °C at 1.5 × 10^−5^ mbar. Crystallinity degrades but is not lost (see Supporting Information, Figure S16). Praseodymium already forms a dense framework at this temperature, as shown in the next chapter (2.2. Thermal conversion processes). The longer the activation under suitable high vacuum conditions, the more pronounced the hysteresis including its horizontal character gets. The horizontal H4-hysteresis can be addressed to slit like pores (see Figure 1). A pore size distribution was calculated using a QSDFT calculation based on a slit-pore model for activated carbon. It starts in the mesoporous region with pore sizes between 5–40 μm for 24 h activation and can be driven to macropores after 96 h reaching pore extensions >200 μm (Figure 1). The presence of micropores plays a lesser role, if the material undergoes the formation of the larger pores, being <10% of the possible microporous surface, as the isotherms indicate. Instead a remarkable mesoporous up to macroporous surface is formed, that reaches surface areas of s_BET_ = 245 m^2^·g^−1^ for a thermal treatment of 96 h.

It is hereby remarkable that this surface modification is bound to the bipy molecules incorporated in the channels of ^2^_∞_[Ln_2_Cl_6_(bipy)_3_]·2bipy. Once removed, no such modification is observed upon treatment under high vacuum, as observed for ^2^_∞_[Sm_3_Cl_9_(bipy)_3_] (**9**) in SEM investigations at comparable conditions.

**Figure 2:**
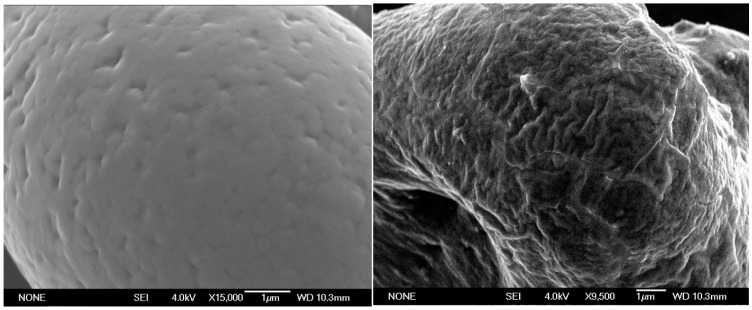
Electron microscopic images (SEM) of${\;}_{\infty }^{2}$[Sm_2_Cl_6_(bipy)_3_]·2bipy (**5**) for an activation of the MOF at 300 °C at 10^−6^ mbar and an activation time of 24 h (**left**) and 96 h (**right**) indicating the ongoing surface modification.

In order to prove the interpretation of slit-pores, samples of${\;}_{\infty }^{2}$[Sm_2_Cl_6_(bipy)_3_]·2bipy (**5**) were also investigated by electron microscopy (SEM) subsequent to equivalent activation times and procedure of the sorption analyses. These investigations corroborate the type-IV adsorption isotherms and show that the formation of larger pores is accompanied by a morphological surface modification. After 24 h at 300 °C and 10^−6^ mbar, the surface of crystals of **5** shows first isotropic holes in the surface. After 96 h at these conditions, the surface is strongly modified and exhibits a system of slit like pores that match with the adsorption experiments (see Figure 2). Thereby, the thermal treatment in combination with suitable vacuum conditions can be used to post-synthetically modify the surface of the MOF particles into a direction of meso- and macropores. The pore size and shape depend on the time scale of this procedure which can thereby be used to modify the pores. X-ray powder diffraction shows that the original compound is retained accompanied only by a slight decrease in crystallinity, which can be expected for such a harsh treatment. Once the bipy molecules are released from the channels no further surface modification is observed. This is corroborated by referring studies on${\;}_{\infty }^{2}$[Sm_2_Cl_6_(bipy)_3_] (**7**) and${\;}_{\infty }^{3}$[Sm_3_Cl_9_(bipy)_3_] (**9**) that do neither contain channel molecules nor show such an effect.

### 2.2. Thermal Conversion Processes

**Figure 3:**
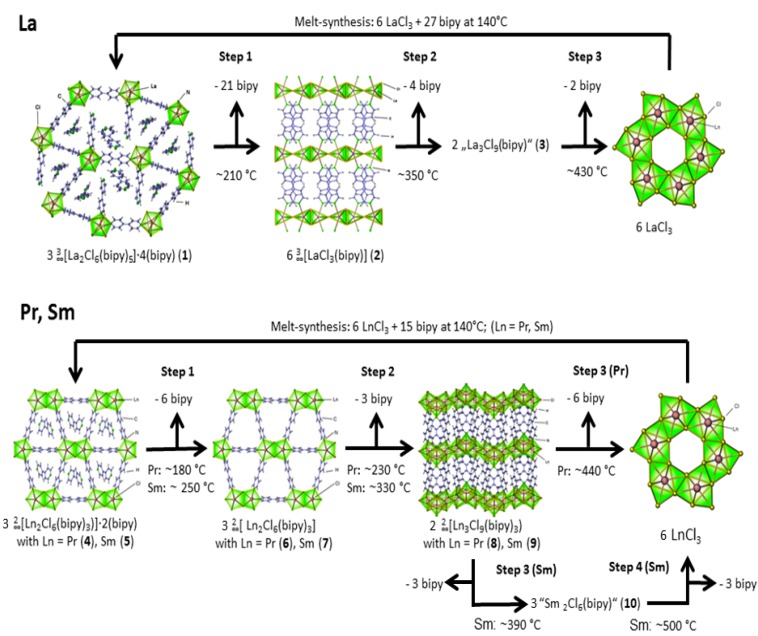
Depiction of the stepwise reaction and thermal condensation processes to new high temperature phases:${\;}_{\infty }^{3}$[La_2_Cl_6_(bipy)_5_]·4bipy (**1**) via${\;}_{\infty }^{3}$[LaCl_3_(bipy)] (**2**) and “La_3_Cl_9_(bipy)” (**3**) to LaCl_3_ (**top**);${\;}_{\infty }^{2}$[Pr_2_Cl_6_(bipy)_3_]·2bipy (**4**) via${\;}_{\infty }^{2}$[Pr_2_Cl_6_(bipy)_3_] (**6**) to${\;}_{\infty }^{2}$[Pr_3_Cl_9_(bipy)_3_] (**8**) and PrCl_3_.${\;}_{\infty }^{2}$[Sm_2_Cl_6_(bipy)_3_]·2bipy (**5**) via${\;}_{\infty }^{2}$[Sm_2_Cl_6_(bipy)_3_] (**7**) to${\;}_{\infty }^{2}$[Sm_3_Cl_9_(bipy)_3_] (**9**) followed by the yet structurally unidentified “Sm_2_Cl_6_(bipy)” (**10**) and SmCl_3_ (**bottom**); see also Supporting Information.

Temperature dependent XRPD and simultaneous DTA/TG investigations allow the in-situ surveillance of reactions of LnCl_3_ and 4,4′-bipyridine forming the initial MOFs${\;}_{\infty }^{3}$[La_2_Cl_6_(bipy)_5_]·4bipy (**1**) [37] and${\;}_{\infty }^{2}$[Ln_2_Cl_6_(bipy)_3_]·2bipy with Ln = Pr (**4**), Sm (**5**) in the molten linker ligand [35,36]. These initial frameworks are hence addressed to as the low-temperature phases (LT), as they are the initial products of these reactions. Upon controlled heating, conversions of these known LT phases to new crystalline high-temperature (HT) phases are observed. The investigations of the thermal properties of these systems allows to explain at which conditions porous structures are available for a certain lanthanide ion and also when and why not (see Figure 3). It becomes evident that activation conditions of 180 °C to 200 °C that are suitable e.g., for the praseodymium MOF are insufficient for samarium, whereas the activation conditions for the Sm-MOF of about 250 °C already initiate formation of a dense framework for Pr, whereas for lanthanum no thermal activation to a porous MOF is possible. A detailed study on the microporosity of the MOF series was reported for Gd in${\;}_{\infty }^{2}$[Ln_2_Cl_6_(bipy)_3_]·2bipy [36].

**Figure 4:**
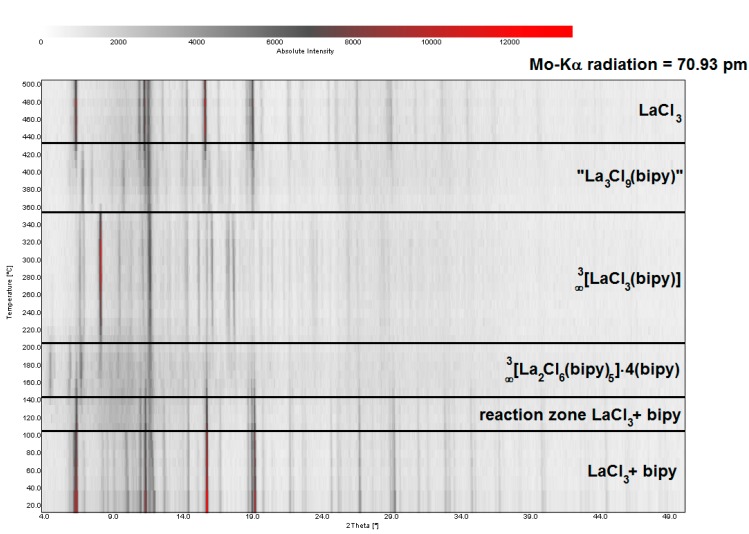
Depiction of temperature-dependent X-ray powder diffraction of the reaction of LaCl_3_ and 4,4′-bipyridine illustrating the temperature dependent formation of several crystalline coordination polymers and MOFs.

**Figure 5:**
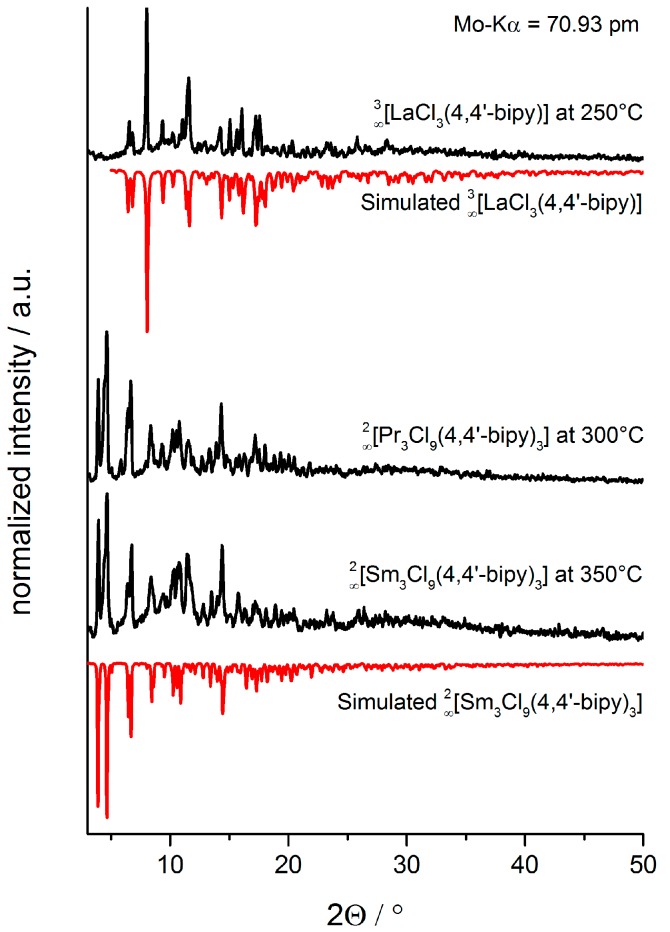
Comparison of the diffraction patterns of **8** and **9** by temperature-dependent XRPD investigations and the simulated diffraction patterns based on single-crystal data.

For the reaction of LaCl_3_ with bipy in a molar ratio of 2 to 9, several defined reaction steps can be identified: subsequent to melting of the ligand bipy at 105 °C, the reaction starts at 140 °C forming${\;}_{\infty }^{3}$[La_2_Cl_6_(bipy)_5_]·4bipy (**1**) (Figure 4). Completeness of the reaction is achieved at about 190 °C, corroborated by removal of the diffraction pattern of LaCl_3_. At 190 °C a slow structural change is observed as the initial framework (now called the low-temperature phase) transforms into the new highly-condensed crystalline frame-work${\;}_{\infty }^{3}$[LaCl_3_(bipy)] (**2**) (step 1, see also Figure 3 and Supporting Information S11). Thereby, 78% of the containing bipy is released by the evaporation of 4 equivalents of intercalated, 2 equivalents of end-on coordinating and one equivalent of bridging bipy. A potentially porous activated network “${“}_{\infty }^{3}$[La_2_Cl_6_(bipy)_5_]” without intercalated bipy molecules cannot be observed. The dimeric La_2_Cl_6_ units are further aggregated to LaCl_3_ sheets coordinated by bipy in a 3D-framework. A direct comparison of the diffraction pattern of the temperature dependent XRPD investigations with the simulated single-crystal data of${\;}_{\infty }^{3}$[LaCl_3_(bipy)] (**2**) shows good accordance (Figure 4 and Figure 5, top). At 350 °C another reaction and structural change occurs as **1** converts to another crystalline and more temperature resistant phase. Simultaneous DTA/TG investigations reveal a split endothermic signal beginning at 330 °C including two mass loss signals with a ratio of 2 to 1, leading to a constitution of “La_3_Cl_9_(bipy)” (**3**) (see also Figure 3, top, step 2). Structural resolution of this phase has not yet been successful. A final slow conversion of “La_3_Cl_9_(bipy)” leads to reformation of LaCl_3_ beginning at 430 °C accompanied by bipy evaporation and carbonization (Figure 3, top, step 3). Accordingly, the melting point of LaCl_3_ can be observed at 820 °C [40]. The reformed LaCl_3_ can now be reacted with bipy again, allowing a cyclic reaction of the reagents through the formation of highly aggregated frameworks and coordination polymers.

**Figure 6:**
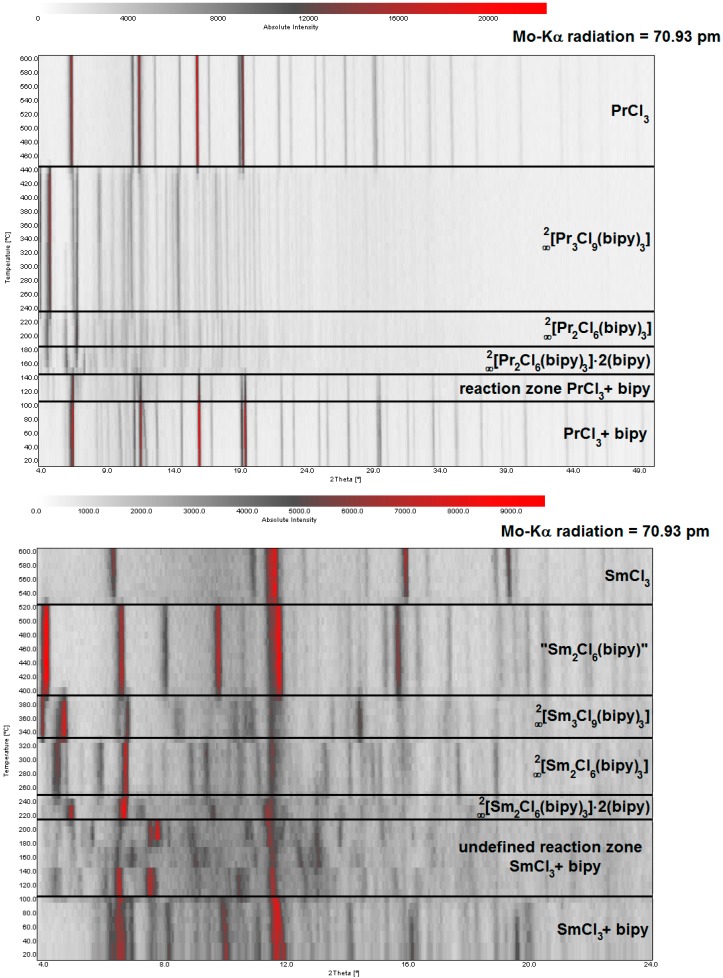
Temperature dependent XRPD investigation of the reaction between PrCl_3_ (**top**)/SmCl_3_ (**bottom**) and 4,4′-bipyridine, illustrating the temperature dependent formation conditions of several coordination polymers and MOFs and the reformation of crystalline LnCl_3_ salts.

The reactions of praseodymium- and samarium-trichloride with 4,4′-bipyridine also exhibit several phases thermally following one another (see Figure 3, bottom, and Figure 6). Initial melting of the ligand bipy can again be observed at 105 °C for both Pr and Sm with temperature dependent XRPD. Reaction of PrCl_3_ or SmCl_3_ and bipy in a molar ratio of 2 to 5 gives the MOFs${\;}_{\infty }^{2}$[Ln_2_Cl_6_(bipy)_3_]·2bipy with Ln = Pr (**4**), Sm (**5**) (Figure 6) as low-temperature phases at 150 °C (Pr) and 210 °C (Sm).

For Sm^3+^ prior to formation of this MOF, a reaction zone with different reflection patterns is observed in the temperature range of 105–210 °C that could not yet be resolved. The two-dimensional networks${\;}_{\infty }^{2}$[Ln_2_Cl_6_(bipy)_3_]·2bipy are thermally activated starting at about 180 °C (Pr) (**4**) and 250 °C (Sm) (**5**), respectively, by removing the two equivalents of intercalated bipy from the cavities of the sheet structure, leading to the porous MOFs ^2^_∞_[Ln_2_Cl_6_(bipy)_3_], with Ln = Pr (**6**), Sm (**7**) (see also Figure 3, bottom, step 1). Possible activation and the resulting microporosity have been reported for the isotypic Gd-MOF, before [36]. To corroborate the findings, here, a sorption study was carried for the Sm containing MOF in **7**. For N_2_ adsorption, isotherms were recorded by the BET method that show an uptake of 168 cm^3^·g^−1^ (77 K) corresponding to a surface of s_BET_ = 648 m^2^·g^−1^ (97% or the Gd-variant) and proving formation of a porous network. This is also in good accordance with the PXRD study of the surface modification study on${\;}_{\infty }^{2}$[Ln_2_Cl_6_(bipy)_3_]·2bipy (see Supporting Information) that indicates retaining of the network structure.

A structural conversion takes place by release of one equivalent of bipy at 230 °C (Pr) and 330 °C (Sm), leading to the new dense sheet-structures${\;}_{\infty }^{2}$[Ln_3_Cl_9_(bipy)_3_] with Ln = Pr (**8**), Sm (**9**) (Figure 3, bottom, step 2). Of **8** and **9**, also single crystals could be grown; a direct comparison of the diffraction patterns of the temperature dependent XRPD investigations with the simulated single-crystal reflection pattern of **8** and **9** shows good accordance (Figure 5). Further aggregation is achieved by connection of the bipy coordinated dimeric Ln_2_Cl_6_ units to strands. These networks are temperature stable up to 440 °C (Pr) and 390 °C (Sm), respectively, as seen in temperature dependent XRPD.

**Figure 7:**
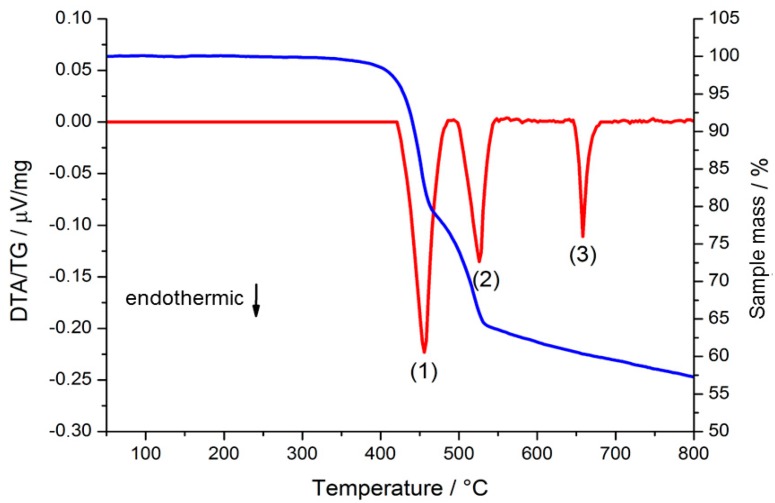
Simultaneous DTA/TG investigations on${\;}_{\infty }^{2}$[Sm_3_Cl_9_(bipy)_3_] (**3**) using a heating rate of 10 °C/min. DTA curve: red; TG curve: blue.

Simultaneous DTA/TG investigations on **8** and **9** corroborate these findings and reveal a mass loss of three (Pr; 34.5%, theor. 38.5%) or 1.5 equivalents (Sm; 20%, theor. 18.9%) of bipy molecules beginning at 350 °C (Pr) and 360 °C (Figure 7, signal 1 and Figure 3, bottom, step 3). Accordingly, the praseodymium and samarium containing reactions behave differently from this step on. The relevant temperatures of XRPD and DTA/TG investigations cannot fully match. Differences in the determined temperatures can be explained by the different analytic methods (closed system for temperature dependent XRPD; open system for DTA/TG investigations). For Sm another high temperature phase can be observed in the range from 390 to 530 °C in temperature dependent XRPD and in the range of 460 to 500 °C in DTA/TG analysis (Figure 7; between signals 1 to 2). A constitution of “Sm_2_Cl_6_(bipy)” (**10**) for the crystalline coordination polymer can be deduced from the mass loss of the TG, which corresponds to the remaining 1.5 equivalents of coordinated bipy (16%, theor. 18.9%) for **9** as starting point of the mass loss (Figure 7, signal 2 and Figure 3, bottom, step 4). For both lanthanides reformation of the crystalline LnCl_3_ salts is observed above 440 °C (Pr) and 530 °C (Sm) in temperature dependent XRPD. Thermal investigations reveal a starting point of the reformation at 350 °C (Pr) and 460 °C (Sm). Furthermore, the melting points of the corresponding LnCl_3_ could be observed: PrCl_3_ at 762 °C (mp. = 769 °C) [41]; SmCl_3_ at 651 °C (mp. = 677 °C) [42]. Analogous to the reaction with LaCl_3_ cyclic reactions of the reagents through the formation of highly aggregated frameworks and coordination polymers are also possible for samarium and praseodymium.

### 2.3. Synthesis of Single Crystalline High-Temperature Phases via a Solvothermal Approach

As described before, the thermal conversion reactions do not lead to products with single crystals suitable for a structure determination. By solvothermal reaction conditions in pyridine, we were able to improve the conditions for crystallization of high-temperature phases, allowing the structural characterization of several of these new phases via single-crystal X-ray determination. Comparison of single crystal data with powder diffraction data reveals identity. However, the use of additional pyridine opens the field of reaction products also to new py containing compounds, as shown for the crystallization of dinuclear complexes [Ln_2_Cl_6_(bipy)(py)_6_], with Ln = Pr, Nd, Sm-Yb [43]. We now show that it can also be used to obtain high-temperature phases in the reaction system LnCl_3_/pyridine, as multiple products can be formed. The solvothermal reaction route with pyridine is an excellent support of the melt reactions as it enables the structural characterization of high-temperature phases and further new products including different aggregations like the one-dimensional chain structure${\;}_{\infty }^{1}$[Ho_2_Cl_6_(bipy)_2_] (**11**). Even *in-situ* generation of new ligands via C-C coupling of bipy to the qtpy ligand is observed, e.g., leading to the two-dimensional network${\;}_{\infty }^{2}$[Gd_2_Cl_6_(qtpy)_2_(bipy)_2_]·bipy (**12**). Qtpy is normally obtained via the C-C coupling of bipy in chloroform reflux at 125 °C using Raney nickel or Pd on charcoal catalysts, [44] which is free of carbonization processes of elder methods with iodine [45] or lithium diisopropylamide. [46] Therefore, the use of GdCl_3_ can lead to catalytic activity in the C-H activation of the ortho-position of bipy leading to a C-C bonding forming small amounts of qtpy, similar to the high temperature reaction of bipy with iodine or LDA; however, it is only a side reaction.

### 2.4. Crystal Structures of${\;}_{\infty }^{3}$[LaCl_3_(bipy)] (**2**),${\;}_{\infty }^{2}$[Ln_3_Cl_9_(bipy)_3_], Ln = Pr (**8**), Sm (**9**),${\;}_{\infty }^{1}$[Ho_2_Cl_6_(bipy)_2_] (**11**), and${\;}_{\infty }^{2}$[Gd_2_Cl_6_(qtpy)_2_(bipy)_2_]·bipy (**12**)

**Figure 8:**
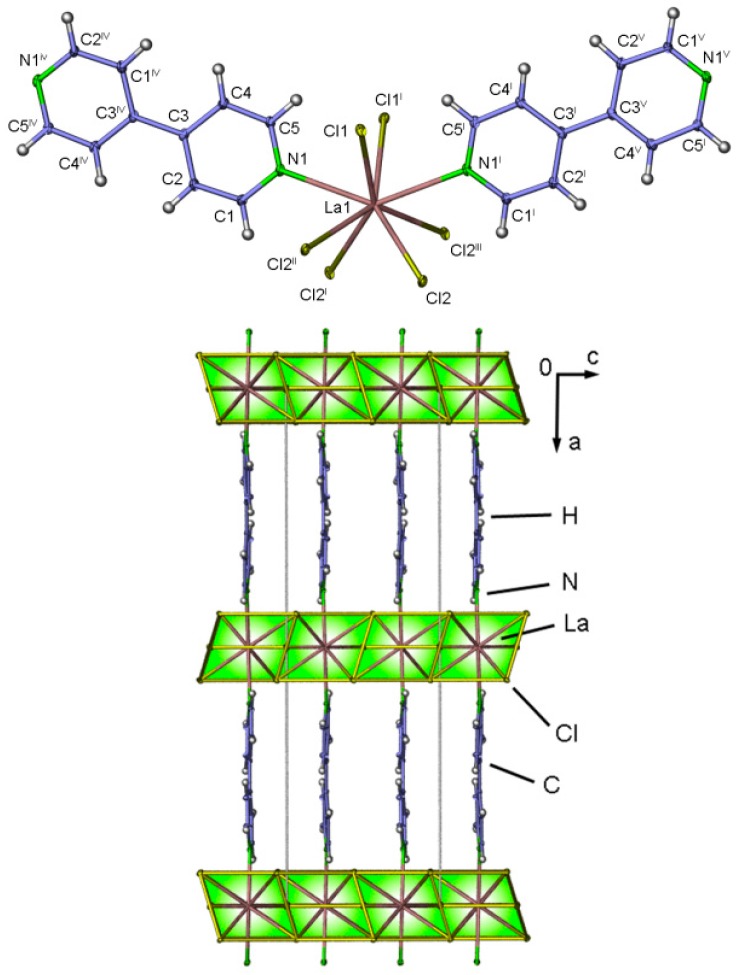
Coordination sphere with complete ligands of${\;}_{\infty }^{3}$[LaCl_3_(bipy)] (**2**) different in two alternating positions of Cl2 within the trigonal-prismatic coordination sphere of the La^3+^ ion. Symmetry operations: ^I^: 1−x, y, ½−z, ^II^: 1−x, 1−y, −z; ^III^: x, 1−y, ½+z; ^IV^: 3/2−x, 2−y, z; ^V^: x−1/2, 2−y, ½−z (**top**). View along the b-axis of the single-crystal structure of${\;}_{\infty }^{3}$[LaCl_3_(bipy)] (**bottom**).

${\;}_{\infty }^{3}$[LaCl_3_(bipy)] (**2**) crystallizes in the orthorhombic space group *Pcca.* Crystallographic data is presented in Table 1, selected interatomic distances can be found in Supporting Information T1. The trivalent La^3+^ ion occupies a single crystallographic position, exhibiting a coordination number of eight representing a distorted trigonal-bicapped prism polyhedron. The coordination sphere consists of six chloride anions located on the trigonal planes of the prism and two nitrogen atoms occupying the bicapped positions (Figure 8). A comparable coordination sphere can be found in LnCl_3_/dinitrile coordination polymers like${\;}_{\infty }^{3}$[LnCl_3_(1,3-Ph(CN)_2_)] with Ln = Eu, Tb [47] and${\;}_{\infty }^{3}$[LnCl_3_(1,4-Ph(CN)_2_)] with Ln = Sm, Gd, Tb [48]. The La^3+^ ion is corner-connected via two single-μ-chloride-ions (La-Cl1-La 180.00(8)°) and edge-connected via two double-μ-chloride bridges (La-Cl2-La 108.85(13)°) forming a Ln-Cl sheet structure along the *bc*-plane (Figure 8, see Supporting Information S1). Comparable angles can be found in the dimeric complex [La_2_Cl_6_(DME)_4_] [49] with 107.28°. The La-Cl distances are in the range of 284.88(4) pm (single-bridge) and 284.62(4)-292.41(7) pm (double-bridge) showing good accordance with the ones observed in ^2^**_∞_**[La_2_Cl_6_(bipy)_5_]·4bipy [37] with 280.6 and 288.7 pm. Furthermore, the angle N-La-N (139.70(13)°), between the back and forth-side coordination of the 4,4′-bipyridine molecules on the Ln-Cl prisms as well as the distance between the LnCl_3_ sheets (1193.7(12) pm) are larger than the ones observed in the related three-dimensional coordination polymer series${\;}_{\infty }^{3}$[LnCl_3_(1,3-Ph(CN)_2_)] with Ln = Eu, Tb [37] and${\;}_{\infty }^{3}$[LnCl_3_(1,4-Ph(CN)_2_)] with Ln = Sm, Gd, Tb. [48] Topological Analysis via TOPOS [50] reveals a point symbol for the La^3+^ ion of {4^8^.6^7^}, a vertex symbol [4.4.4.4.4.4.4.4.6(2).*.*.*] and ring types [4a.4a.4a.4a.4b.4b.4b.4b.6c(2).*.*.*]. The point symbol for the uninodal 6-c net is {4^8^.6^7^} and the three-dimensional network topology type is sxa (See Supporting Information S2). Similar topology can be found in MOFs like${\;}_{\infty }^{3}$[Zn_4_([4-(carboxyphenyl)oxamethyl]-methaneacid)(H_2_O)_3_(DMA)]·2H_2_O [51] or in lanthanide containing compounds like${\;}_{\infty }^{3}$[LnCl_3_(1,3-Ph(CN)_2_)] with Ln = Eu, Tb [47] and${\;}_{\infty }^{3}$[LnCl_3_(1,4-Ph(CN)_2_)] with Ln = Sm, Gd, Tb [48].

**Table 1:** Crystallographic data for${\;}_{\infty }^{3}$[LaCl_3_(bipy)] (**2**),${\;}_{\infty }^{2}$[Ln_3_Cl_9_(bipy)_3_] with Ln = Pr (**8**), Sm (**9**),${\;}_{\infty }^{1}$[Ho_2_Cl_6_(bipy)_2_] (**11**),${\;}_{\infty }^{2}$[Gd_2_Cl_6_(bipy)_2_(bipy)_2_]·bipy] (**12**).

	${\;}_{\infty }^{3}$[LaCl_3_(bipy)] (2)	${\;}_{\infty }^{2}$[Pr_3_Cl_9_(bipy)_3_] (8)	${\;}_{\infty }^{2}$[Sm_3_Cl_9_(bipy)_3_] (9)	${\;}_{\infty }^{1}$[Ho_2_Cl_6_(bipy)_2_] (11)	${\;}_{\infty }^{2}$[Gd_2_Cl_6_(qtpy)_2_ (bipy)_2_]·bipy (12)
Formula weight/g·mol^−1^	401.44	1210.36	1238.65	854.95	1608.40
Crystal system	orthorhombic	orthorhombic	orthorhombic	monoclinic	monoclinic
Space group	*Pcca*	*Cmcm*	*Cmcm*	*P*2_1_/*c*	*C*2/*m*
*a*/pm	2387.4(5)	1213.49(14)	1206.99(5)	988.4(2)	1177.53(13)
*b*/pm	744.9(2)	2015.5(2)	2015.93(9)	2463.1(6)	2351.7(2)
*c*/pm	717.8(2)	1606.2(2)	1590.81(7)	1137.2(3)	1687.2 (2)
β/°	-	-	-	112.142(7)	107.710(3)
Volume/pm^3^	1276.6(4)	3928.5(8)·10^6^	3870.8(3)·10^6^	2564.4(11)·10^6^	4450.8(8)·10^6^
Z	4				
*d*_c_/g/cm^3^	2.089	2.0463	2.126	2.214	2.400
Diffractometer	Bruker Apex II	SMART Bruker Apex	Bruker Apex II	Bruker Apex II	Bruker Apex II
Monochromator	Helios-mirror	Graphite	Helios-mirror	Helios-mirror	Helios-mirror
Radiation	70.93 pm (Mo-Kα)				
Temperature/K	100(3)	168(5)	100(3)	100(3)	100(3)
2θ range	3.42 ≤ 2θ ≤ 60.7°	3.92 ≤ 2θ ≤ 60.08°	3.94 ≤ 2θ ≤ 60.22°	4.2 ≤ 2θ ≤ 61.82°	2.54 ≤ 2θ ≤ 60.18°
μ/mm^−1^	3.946	4.305	5.144	6.768	1.697
F_000_	761.5	2308.8	2340.0	1604.0	1584.0
Reflections collected	17347	22073	28523	39177	33891
Independent reflections	1834	3091	3023	7326	6484
	[R(int) = 0.0355]	[R(int) = 0.1876]	[R(int) = 0.0768]	[R(int) = 0.1042]	[R(int) = 0.2972]
Data/restraints/parameters	1834/0/74	3091/0/122	3023/0/123	7326/0/288	6484/0/225
*S*	1.143	1.001	1.077	1.018	1.083
*R*_1_ for n reflections > [I ≥ 2σ (I)] ^[a]^	0.0255	0.0580	0.0356	0.0432	0.1241 ^[a]^
*R*_1_[all data] ^[a]^	0.0372	0.0993	0.0541	0.0915	0.3063 ^[a]^
*wR*_2_[all data] ^[b]^	0.0458	0.1341	0.0700	0.0789	0.3879 ^[b]^
Largest diff. peak/hole/(e pm^−3^)·10^−6^	1.02/−1.33	2.78/−3.25	2.53/−1.08	3.69/−4.07	2.00/−2.05

^[a]^ R_1_ = ∑[|F_o_|-|F_c_|]/∑| F_o_|; ^[b]^ wR_2_ = [∑ w (|F_o_|^2^-|F_c_|^2^)^2^ /∑ w (|F_o_|^2^)^2^]^1/2^.

**Figure 9:**
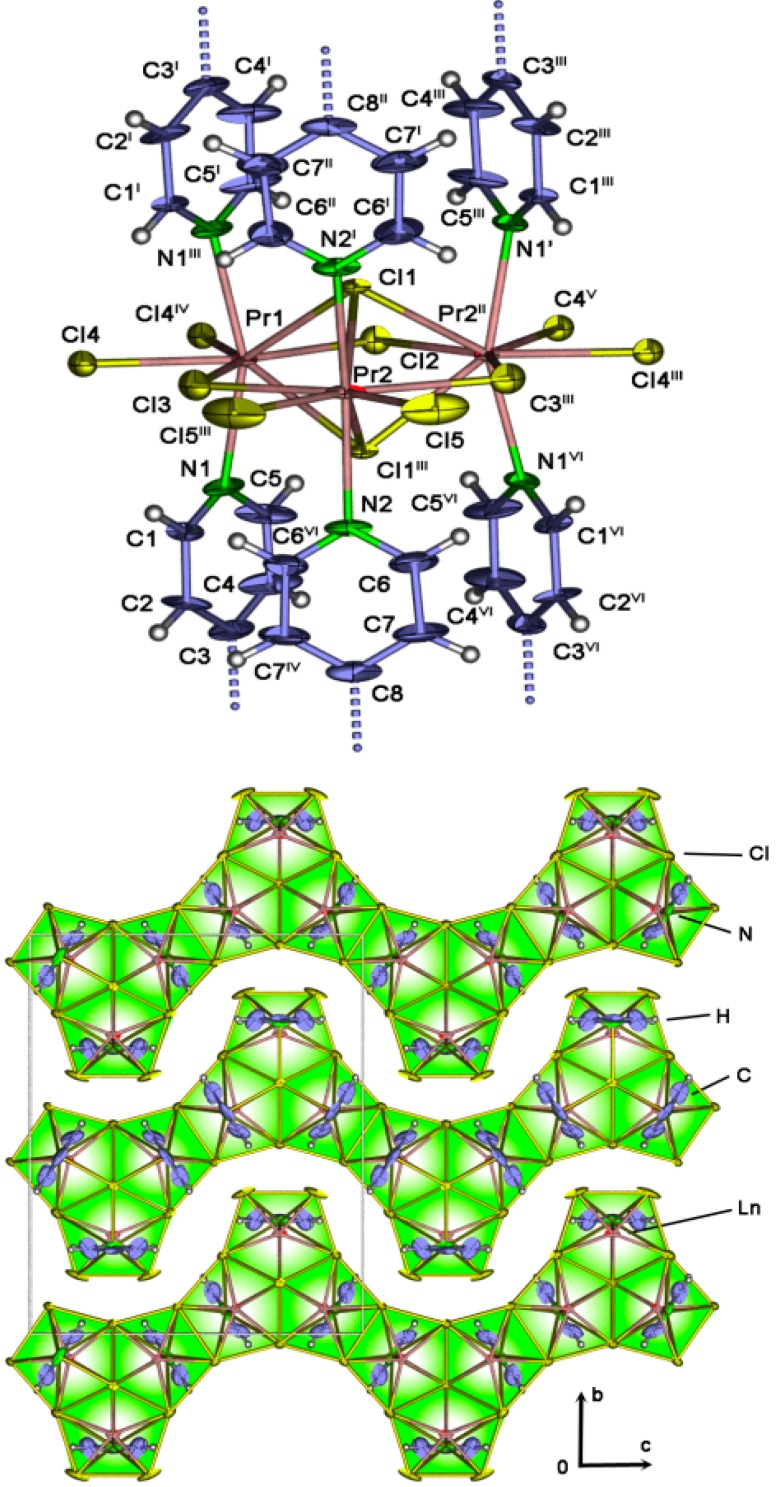
The cluster-like arrangement in${\;}_{\infty }^{2}$[Pr_3_Cl_9_(bipy)_3_], H-atoms are omitted for clarity; symmetry operations: ^I^ −x, y, z; ^II^ −x, y, 3/2−z; ^III^ −x, y, 3/2−z; ^IV^ x, −y, 2−z; ^V^ −x, −y, z−1/2; ^VI^ x, y, 3/2−z (**top**). View along the a-axis of the crystal-structure of${\;}_{\infty }^{2}$[Ln_3_Cl_9_(bipy)_3_] for Ln = Pr (**8**) (**bottom**); thermal ellipsoids are depicted with 50% probability.

${\;}_{\infty }^{2}$[Ln_3_Cl_9_(bipy)_3_] with Ln = Pr (**8**), Sm (**9**) crystallize in the orthorhombic space group *Cmcm*. Crystallographic data is also presented in Table 1, selected interatomic distances can be found in the Supporting Information T2. The structure consists of two Ln^3+^ ions on independent crystallographic sites for Pr in **8** and Sm in **9**, respectively (Figure 9). Both ions exhibit a coordination number of eight, resulting in dodecahedral coordination spheres. The coordination sphere of Ln1 consists of four μ-bridging chloride ions, two μ_3_-bridging chloride ions and two nitrogen atoms of bipy. Ln2 is surrounded by two terminal chloride ions, two μ-bridging chloride ions and two μ_3_-bridging chloride ions next to two nitrogen atoms of bipy. The Ln-Cl distances for the terminally bonded Cl-ions are 266.4(2) pm for Pr(**2**) and 266.6(2) pm for Sm(**3**). The μ-bridging Cl-ligands are in the range of 276.9(3)–291.3(3) pm for Pr(**2**) and 273.17(15)–289.6(2) pm for Sm(**3**). For the μ_3_-bridged chloride atoms Ln-Cl distances are in the range of 296.6(2)–296.4(2) pm (Pr(**2**)) and 292.54(14)–293.22(10) pm. Interatomic distances and angles correspond to the MOFs${\;}_{\infty }^{2}$[Ln_2_Cl_6_(bipy)_3_]·2bipy, Ln = Pr (**4**), Sm (**5**), and the dinuclear complex [Sm_2_Cl_6_(bipy)(py)_6_] [52]. The structural motif formed by the μ_3_-bridging chloride atoms connecting the Ln^3+^ ions can be described with a trimeric Ln_3_Cl_9_ cluster-like arrangement. This structural motif is known from the isolated anionic cluster complex [Mo_3_Br_11_]^2−^. [53] The Ln_3_Cl_9_ cluster-like arrangements are connected via μ-edge-connected double halide bridges in a by 180° alternating way, forming a one-dimensional Ln-Cl strand. The sequence of the alternating trimeric Ln-Cl clusters can also be observed in the one-dimensional strand structure [(C_6_H_5_Me)Sm(AlCl_4−x_I_x_)_2_]_n_ [54]. The Ln-Cl strands are parallel to the *c*-axis with an offset of half *a* and *b*-axes, and are connected via coordinating μ-bridging bipy molecules to a sheet structure parallel to the *ac*-plane (Figure 9, see Supporting Information S3 and S4). The barycentre distance between the single sheets is half a *b*-axis with 1007.75(2) pm for Pr (**8**) and 1007.96(9) pm for Sm (**9**). The overall structure is a dense sheet-structure with no intercalated solvent molecules. Topology analysis reveals a novel unknown topology type. Ln1 can be described with the point symbol {3.4^6^.6^3^}, the vertex-symbol [3.4.4.4.4.4.4.*.*.*] and the ring types are [3a.4a.4a.4b.4b.4c.4c.*.*.*]. Ln2 can be described with the point symbol {3.4^4^.6}, the vertex-symbol [3.*.4.4.4.4] and the ring types are [3a.*.4b.4b.4b.4b]. The point symbol for the 2-nodal 4,5-c net is {3.4^4^.6}{3.4^6^.6^3^}2 (See Supporting Information S5).

**Figure 10:**
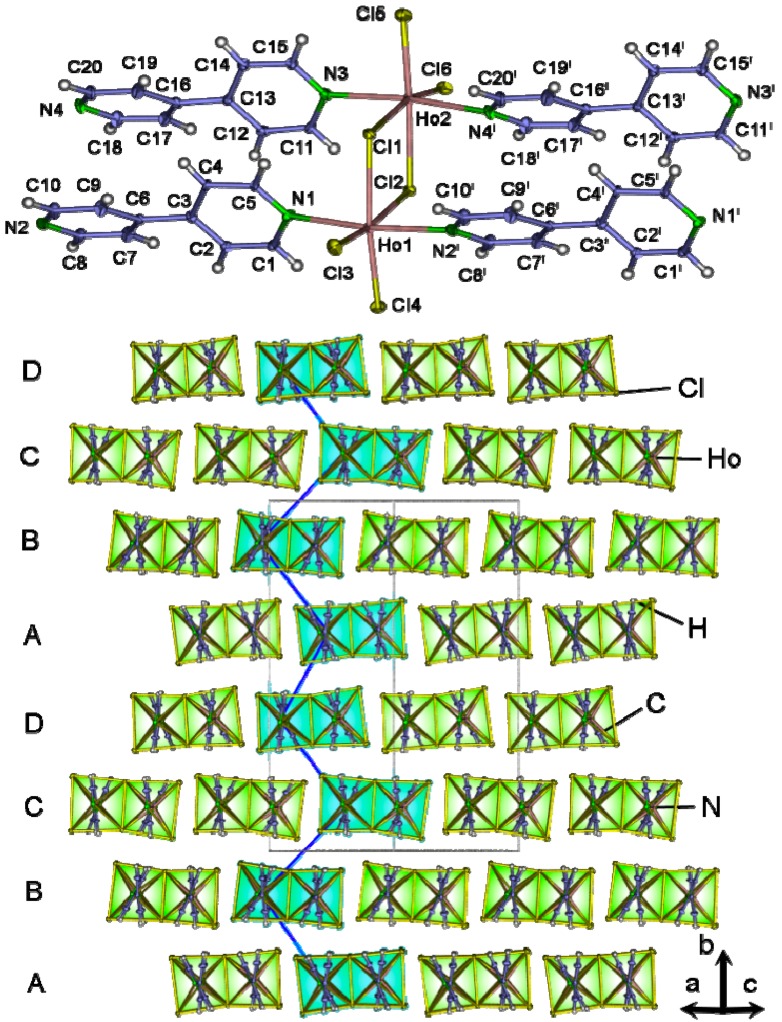
Extended coordination sphere with ligands of${\;}_{\infty }^{1}$[Ho_2_Cl_6_(bipy)_2_] (**11**); thermal ellipsoids are depicted with 50% probability; symmetry operators: ^I^ 1+x, +y, −1+z (**top**). View along [101] parallel to the ladder like strands of${\;}_{\infty }^{1}$[Ho_2_Cl_6_(bipy)_2_]; the stacking order is marked in blue (**bottom**).

The compound${\;}_{\infty }^{1}$[Ho_2_Cl_6_(bipy)_2_] (**11**) crystallizes in the monoclinic space group *P*2_1_/*c*. Crystallographic data is presented in Table 1, selected interatomic distances in Supporting Information T3. Ho^3+^ ions occupy two independent crystallographic sites and are both octahedrally coordinated by four equatorial Cl-ions and two axial N-atoms (Figure 10). The Ho-Cl(terminal) distances 250.3(2)–254.0(2) pm and Ho-N distances 243.5(5)–245.7(5) pm can be compared with the ones observed in the coordination polymer${\;}_{\infty }^{1}$[Ho(glu)(phen)Cl] [55] with Ho-Cl 253.3–264.71 pm and Ho-N 242.9–245.6 pm. Both distorted octahedrons are edge-connected on the equatorial plane via a μ-double-chloride bridge. Ho-Cl distances are in the range of 269.5(2)–270.5(2) pm, comparable to [[Zr_2_(O^i^Pr)_9_]HoCl_2_]_2_ [56] with Ho-Cl in the range of 272.9–273.1 pm. The angles N-Ho-N with 172.7(2)° and 172.3(2)° are differing from 180° for an ideal octahedron accompanied by the Cl-Ln-Cl angles within the equatorial plane differing from ideal 90° to 103.49(5) to 79.09(4)°. Both octahedrons form a dimeric-octahedral SBU (secondary building unit) Ho_2_Cl_6_N_4_. Such a SBU can e.g., be found in the dinuclear thallium complex [TlCl_3_(nica)_2_]·2.2(nica) [57]. The dimeric units are connected via bipy to a ladder like double strand along the direction [101] (Figure 10, see Supporting Information S6 and S7). The double strands are superposable to every fourth chain along their length axis within the *ac*-plane. The view along the diagonal [101] reveals a packing sequence of ABCD along the b-axis until translational coincidence. Investigations via TOPOS [50] reveal a point symbol for both Ho^3+^ ions and the double strand of {4^2^.6}, with a vertex-symbol of [4.4.*] and [4a.4a.*] ring types. The four edge circulation via a lanthanide double-chloride μ-bridge and bipy can also be found in the complexes [{MCl(Cp*)}_4_}_2_(bipy)_2_]·4(CF_3_SO_3_) with M = Ir, Rh [58] and [(Cp*^t^*Rh)_4_(μ-Cl)_4_(bipy)_2_]·4(OTf) [59]. The uninodal 3-c, SP-1 net topology type (see Supporting Information S8) was e.g., observed in the isotopological compound [Fe_2_L(dpa)_2_]·× (MeOH) (L = Tetra-ethyl-[2,2′,2′′,2′′′]-[1,2,4,5-phenylentetra(iminomethylidyn)]tetra(3-oxobutanato)]) [60].

**Figure 11:**
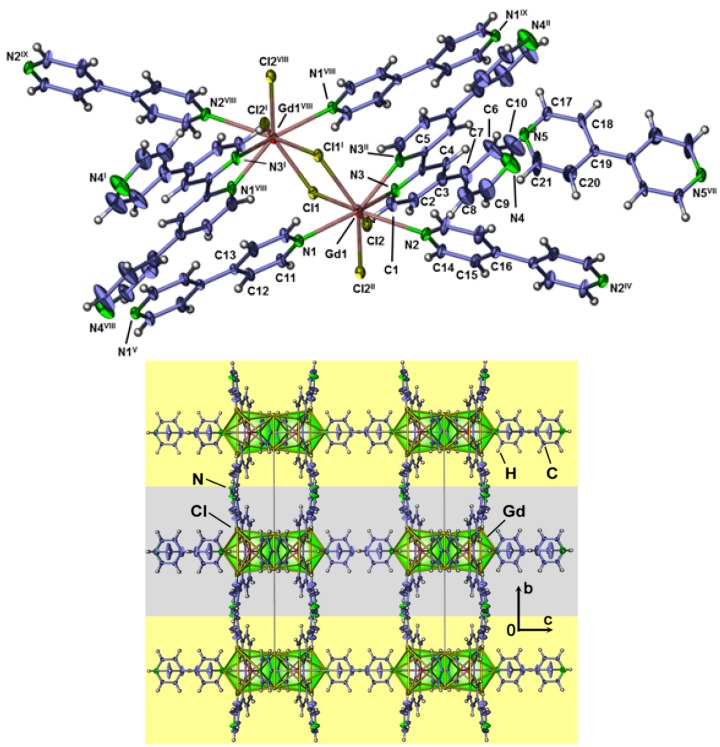
Extended coordination sphere with complete ligands of${\;}_{\infty }^{2}$[Gd_2_Cl_6_(qtpy)_2_(bipy)_2_]·bipy (**12**); thermal ellipsoids are depicted with 30% probability. Symmetry operations: ^I^: 1−x, 1−y, −z; ^II^: x, 1−y, z; ^III^: 1−x, y, 1−z; ^IV^: 1−x, 1−y, 1−z; ^V^: 2−x, y, −z; ^VI^: 2−x, 1−y, −z; ^VII^: x, y, 1−z; ^VIII^: 1−x, y, −z; ^IX^: x−1, 1−y, z; (**top**). View along the *a*-axis of the single-crystal structure of${\;}_{\infty }^{2}$[Gd_2_Cl_6_(qtpy)_2_(bipy)_2_]·bipy with parallel sheets marked in yellow and grey (**bottom**).

${\;}_{\infty }^{2}$[Gd_2_Cl_6_(qtpy)_2_(bipy)_2_]·bipy (**12**) crystallizes in the monoclinic space group *C*2/m. Further crystallographic data is presented in Table 1, selected interatomic distances can again be found in the Supporting Information T4. The Gd^3+^ ion is coordinated by three chloride anions and four nitrogen atoms forming a distorted bi-capped, trigonal-prismatic polyhedron with a coordination number of eight (Figure 11). The terminal Gd-Cl distance (265.8(4) pm) and the μ-bridging Gd-Cl distances (279.4(4) pm) are comparable to [GdCl(μ-Cl)_2_(H_2_O)_2_CH_3_CN] [61] with 267.1 pm (terminal-Cl) and 281.7–283.8 pm (μ-bridging Cl). The Gd-N distances in **12** are 267(2)–279(2) pm for the N-atoms of bridging bipy and 254.8 pm for the chelating qtpy. Similar distances can be found in the compounds [Gd(C_8_H_7_O_2_)_3_]·0.5(bipy) [62] with 264.0 pm and 253.8–256.9 pm in${\;}_{\infty }^{1}$[Cr(CN)_4_(μ-CN)_2_-Gd(H_2_O)_4_(2,2′-bipy)]·4(H_2_O)·1.5(2,2′-bipy) [63]. The distorted trigonal prisms are edge-connected via a double-chloride bridge, forming a dimeric Ln_2_Cl_6_ cluster with a Gd-Cl-Gd angle of 107.22(3)°. The bi-capped positions are occupied by N-atoms of μ-bridging 4,4′-bipy molecules, linking the dimeric Ln_2_Cl_6_ clusters to a two dimensional network. Furthermore, each Gd^3+^ ion in the dimeric clusters is coordinated by the *in-situ* formed ligand qtpy (4,4′:2′,2′′:4′′,4′′′-quaterpyridine). No further bridging coordination is observed. Similar coordination of qtpy can be found in the photoactive Ru/Re-compounds [Ru(qtpy)(phen)][PF_6_]_2_·benzene [64] and [Re_2_Cl_2_(CO)_6_Ru_2_(qtpy)_2_(phen)_4_][PF_6_]_4_·1.13(CH_3_NO_2_)·1.24(H_2_O) [65]. The cavities within the sheets are of rhomboid shape and are filled with intercalated bipy. The sheets are stacked along b-axis in an alternating way (Figure 11, see Supporting Information S9 and S10). The space between the sheets is occupied by py or bipy molecules [66]. As the crystals were picked out of a decomposition process with strongly carbonized products, the amount of intercalated pyridine and bipy could not be determined via bulk-analytics. Investigations of the topology lead to a point symbol for Gd of {6^3^} with a vertex symbol of [6.6.6] [50]. The point symbol for the uninodal 3-c net is {6^3^} and the topology type is *hcb* (see Supporting Information S11). The same topology was e.g., found in the sheet structures of${\;}_{\infty }^{2}$[Ni(deen)(N_3_)_2_] [67] or${\;}_{\infty }^{2}$[Ln(HBIDC) (ox)_0.5_(H_2_O)_3_] with Ln = Tb, Ho [68].

The structural data acquired by single-crystal X-ray structure determination was verified by powder X-ray investigations on the reaction products of **2**, **8**–**9** synthesized via solvothermal approach (see also Supporting Information S12–S14). XRPD-investigations of the bulk products of **11**–**12** show no diffraction patterns due to decomposition and amorphous bulk products.

### 2.5. Photoluminescence and Vibrational Spectroscopy

**Figure 12:**
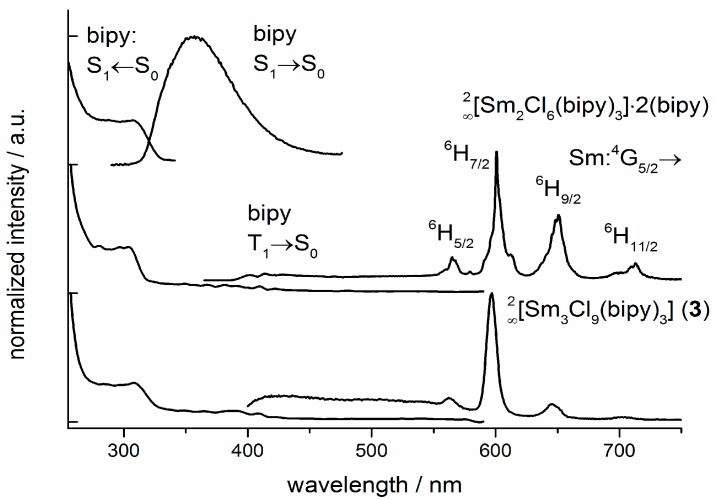
Photoluminescence investigation on the ligand bipy and the Sm-MOF${\;}_{\infty }^{2}$[Sm_2_Cl_6_(bipy)_3_]·2(bipy) (**5**) and${\;}_{\infty }^{2}$[Sm_3_Cl_9_(bipy)_3_] (**9**) in solid state at room temperature. Emission spectra were recorded at λ_exc_ = 300 nm, excitation spectra at the maximum of the referring emission.

The photoluminescence of the MOF series${\;}_{\infty }^{2}$[Ln_2_Cl_6_(bipy)_3_]·2bipy has been already elaborated for Eu^3+^ and Tb^3+^ including chromaticity control via statistic replacement of both ions [35,36]. Further photoluminescence properties can be observed for the MOF${\;}_{\infty }^{2}$[Sm_2_Cl_6_(bipy)_3_]·2bipy (**5**) and for the condensed high-temperature phase${\;}_{\infty }^{2}$[Sm_3_Cl_9_(bipy)_3_] (**9**) (Figure 12). The salmon-red luminescence of both compounds is Sm^3+^ centred and caused by 4*f*-4*f* transitions from the excited state ^4^G_5/2_ to the states ^6^H_J_ with *J* = 5/2–11/2. The broad excitation band in the UV-range with the two maxima λ < 250 nm and λ = 300 nm can be correlated to the excitation of bipy from its ground state S_0_ to the excited singulet state S_1_. The spin-orbit coupling of the Sm^3+^-ion causes an intersystem crossing of the excitation energy from singlet S_1_ state to the triplet levels in both Sm^3+^ containing compounds. Furthermore, the linker bipy and Sm^3+^ show an antenna effect [69,70] by transferring energy from the excited triplet level to excited 4f-states of Sm^3+^ at higher energies than the emissive ^4^G_5/2_ state. The excitation energy then moves via internal conversion to the emissive level of Sm^3+^ causing light emission with four different emission wavelengths (λ_Emission_ = 566, 602, 649, 708 nm). The emissive triplet state can also be identified on the vibrational coupling of broad band emission in the range of λ = 400–420 nm in the Sm^3+^-containing MOF. 

Similar luminescence effects based on antenna effect sensitized Sm^3+^ emission can be seen in various Sm^3+^ containing complexes [12,71] and frameworks [9,13]. Thereby, the overall efficiency of the energy transfer and quantum yield is lower than in compounds containing Eu^3+^ or Tb^3+^ luminescence centers [72]. The Mid-IR spectra of the products confirm the coordination of bipy to the Ln^3+^-ions due to the shift of the ν(C=N) ring vibration from 1591 cm^−1^ for the free molecule [73] to 1602 cm^−1^ La (**2**), 1603 cm^−1^ Pr (**8**) and 1606 cm^−1^ for Sm (**9**) for the metal-coordinating molecule [74]. Furthermore, the IR-bands at 1487 cm^−1^ La (**2**) and 1489 cm^−1^ Sm (**9**) can be identified as metal-bonded pyridine molecules [75], indicating the presence of new py-containing compounds as free pyridine has its vibrational band at 1482 cm^−1^ (for symmetric in plane ring vibrations) [76,77].

## 3. Experimental Section

All experiments were carried out under inert conditions (Argon atmosphere) using vacuum line, Schlenk and glovebox (LabMaster SP, MBraun GmbH, Garching, Germany and Pure Lab, Innovative Technology, Amesbury, MA, USA) as well as DURAN™ ampoule techniques. Heating furnaces based on Al_2_O_3_ tubes with resistance heating and NiCr/Ni temperature elements with Eurotherm 2416 control units were used. The anhydrous rare earth chlorides were prepared by the published ammonium halide route [78] using the oxides La_2_O_3_ (99.9%, RC-Nukor, Phoenix, AZ, USA), Sm_2_O_3_ (99.9%, Auer-Remy GmbH, Hamburg, Germany), Pr_6_O_11_ (99.9%, Serva GmbH, Heidelberg, Germany), Gd_2_O_3_ (99.9%, Koch Chemicals LTD, Hertford, England, UK), Ho_2_O_3_ (99.9%, Strem, Newburyport, MA, USA), HCl solution (10 mol·L^−1^, reagent grade) and NH_4_Cl (99.9%, Fluka, Buchs, SG, Switzerland). The trivalent lanthanide ammonium chlorides were decomposed to LnCl_3_ and subsequently purified by sublimation under vacuum at elevated temperature. 4,4′-bipyridine (98%, Acros-Organics, Geel, Belgium) was dried under vacuum, and anhydrous pyridine (99%, Acros-Organics, Geel, Belgium) was used as purchased.

### 3.1. Synthesis

Rare earth chlorides LnCl_3_, 4,4′-bipyridine and pyridine were sealed in evacuated (3 × 10^−3^ mbar) glass ampoules using Quickfit techniques and frozen with liquid nitrogen. The 4,4′-bipyridine was placed on the bottom of the ampoule, while the corresponding LnCl_3_ was overlaid on top of the organic ligand. Pyridine was slowly poured on the reagents, moistening the LnCl_3_. The reaction ampoules were vertically placed into the heating device.

#### 3.1.1. Synthesis of${\;}_{\infty }^{3}$[La_2_Cl_6_(bipy)_5_]·4(bipy) (**1**),${\;}_{\infty }^{2}$[Ln_2_Cl_6_(bipy)_3_]·2(bipy), Ln = Pr (**4**), Sm (**5**), and${\;}_{\infty }^{2}$[Ln_2_Cl_6_(bipy)_3_], Ln = Pr (**6**), Sm (**7**), Eu (**13**), Eu/Tb (**14**)

${\;}_{\infty }^{3}$[La_2_Cl_6_(bipy)_5_]·4(bipy) and${\;}_{\infty }^{2}$[Ln_2_Cl_6_(bipy)_3_]·2(bipy), Ln = Pr, Sm, Eu/Tb were synthesized from LnCl_3_ and 4,4′-bipyridine according to references [25] and [26]. The thermal activation giving the microporous MOFs${\;}_{\infty }^{2}$[Ln_2_Cl_6_(bipy)_3_] were carried out according to reference [27].

#### 3.1.2. Synthesis of${\;}_{\infty }^{3}$[LaCl_3_(bipy)] (**2**)

LaCl_3_ (0.3 mmol = 74 mg), 4,4′-bipyridine (C_10_H_8_N_2_, 1.2 mmol = 187 mg) and pyridine (C_5_H_5_N, 1.2 mmol = 95 mg) were transferred to an ampoule and treated as described above. A subsequent heating program was applied consisting of five steps starting with heating to 120 °C at a rate of 10 °C·h^−1^, followed by additional heating to 300 °C at 2 °C·h^−1^. The temperature was maintained for 96 h then lowered to 120 °C at 2 °C·h^−1^ and to room temperature at 1 °C·h^−1^. The reaction yielded a colorless reaction product on the bottom of the ampoule. Compound **2** was washed with anhydrous pyridine (2 × 2 mL) and dried in vacuum. The product is air and moisture sensitive. Yield: 90 mg (46%).

Vibrational spectroscopy of${\;}_{\infty }^{3}$[LaCl_3_(bipy)] MIR (KBr): ν(C-H) 3074 w, ν(C-N ring) 1634 w, 1602 vs, ν(C-C ring) 1530 vs, 1415 vs, 1131 w, 1057 s, δ(C-H) 1487 s, 1319 m, 1225 vs, ν(C-C, aromatic) 997 s, γ(C-H) 957 m, 831 w, δ(C=C, aromatic) 1043 m, 797 vs, 726 s, 675 m, 620 vs, 519, γ(C=C, aromatic) 457 m·cm^−1^.${\;}_{\infty }^{3}$[LaCl_3_(bipy)] (401.44 g·mol^−1^); C 30.20 (calc. 29.91); H 2.06 (2.01); N 6.50 (6.98)%. An amount of unreacted LaCl_3_ was determined as 38% by refinement from Rietveld analysis in corroboration with the elemental analysis of the bulk product and the reaction yield.

#### 3.1.3. Synthesis of${\;}_{\infty }^{2}$[Pr_3_Cl_9_(bipy)_3_] (**8**)

PrCl_3_ (0.3 mmol = 74 mg), 4,4′-bipyridine (C_10_H_8_N_2_, 1.2 mmol = 187 mg) and pyridine (C_5_H_5_N, 1.2 mmol = 95 mg) were transferred to an ampoule and treated as described above. The heating program consisted of five steps starting with heating to 120 °C at 10 °C·h^−1^, followed by additional heating to 250 °C at 2 C·h^−1^. The temperature was maintained for 100 h then lowered to 120 °C at 2 °C·h^−1^ and to room temperature at 1 °C·h^−1^. The reaction yielded a green, crystalline reaction product on the bottom of the ampoule. The compound was washed with anhydrous pyridine (2 × 2 mL) and dried in vacuum. The product is air and moisture sensitive. Yield: 85 mg (70%).

Vibrational spectroscopy of${\;}_{\infty }^{3}$[Pr_3_Cl_9_(bipy)] MIR (KBr): ν(C-H) 3055 m, ν(C-N ring) 1603 vs, ν(C-C ring) 1533 m, 1489 m, 1070 s, δ(C-H) 1442 m, 1321 vw, 1223 s, 1153 vw, ν(C-C, aromatic) 1003 s, γ(C-H) 852 vw, 805 vs, 760 m, 729 w, δ(C=C, aromatic) 1041 m, 705 m, 681 vw, 626 vs, 480 m·cm^−1^.

#### 3.1.4. Synthesis of${\;}_{\infty }^{2}$[Sm_3_Cl_9_(bipy)_3_] (**9**)

SmCl_3_ (0.4 mmol = 103 mg), 4,4′-bipyridine (C_10_H_8_N_2_, 0.8 mmol = 125 mg) and pyridine (C_5_H_5_N, 0.8 mmol = 76 mg) were transferred to an ampoule and prepared as described above. The heating program consisted of five steps starting with heating to 90 °C at 10 °C·h^−1^, followed by additional heating to 310 °C at 2 C·h^−1^. The temperature was maintained for 72 h then lowered to 90 °C at 5 °C·h^−1^ and to room temperature at 10 °C·h^−1^. The reaction yielded a colourless, crystalline reaction product on the bottom of the ampoule. The compound was washed with anhydrous pyridine (2 × 2 mL), to remove excess 4,4′-bipyridine and dried in vacuum. The product is air and moisture sensitive. Yield: 105 mg (65%).

Vibrational spectroscopy${\;}_{\infty }^{2}$[Sm_3_Cl_9_(bipy)_3_] MIR (KBr): ν(C-H) 3056 m, ν(C-C, ring) 1606 vs, ν(C-C/N aromatic) 1533 m, 92 m, 1417 s, 1071 s, δ(C-H) 1329 vw, 1224 m, ν(C-C, aromatic) 1004 s, γ(C-H) 805 vs, 7 w, δ(C=C, aromatic) 1043 m, 676 vw, 628 s, 474 m·cm^−1^. ^2^_∞_[Sm_3_Cl_9_(bipy)_3_] (1238.72 g·mol^−1^); C 28.74 (calc. 29.09); H 2.15 (1.95); N 6.71 (6.78)%

#### 3.1.5. Synthesis of${\;}_{\infty }^{1}$[Ho_2_Cl_6_(bipy)_2_] (**11**)

HoCl_3_ (0.3 mmol = 78 mg), 4,4′-bipyridine (C_10_H_8_N_2_, 0.6 mmol = 94 mg) and pyridine (C_5_H_5_N, 0.3 mmol = 24 mg) were transferred to an ampoule and prepared as described above. The heating program consisted of five steps starting with heating to 120 °C at 10 °C·h^−1^, followed by additional heating to 320 °C at 5 °C·h^−1^. The temperature was maintained for 48 h then lowered to 120 °C at 2 °C·h^−1^ and to room temperature at 5 °C·h^−1^. The reaction yielded a black carbonized product. After washing with pyridine (2 × 2 mL), single-crystals could be picked for single-crystal X-ray determination. As the phase is only a side product accompanying the decomposition/carbonization of the organic ligands, no suitable elemental analysis and IR could be achieved. The product is air and moisture sensitive.

#### 3.1.6. Synthesis of${\;}_{\infty }^{2}$[Gd_2_Cl_6_(qpy)_2_(bipy)_2_]·bipy (**12**)

GdCl_3_ (0.4 mmol = 105 mg), 4,4′-bipyridine (C_10_H_8_N_2_, 2.0 mmol = 312 mg) and pyridine (C_5_H_5_N, 0.8 mmol = 63 mg) were transferred to an ampoule and prepared as described above. The heating program consisted of five steps starting with heating to 120 °C at 10 °C·h^−1^, followed by additional heating to 300 °C at 3 °C·h^−1^. The temperature was maintained for 168 h then lowered to 120 °C at 3 °C·h^−1^ and to room temperature at 2 °C·h^−1^. The reaction yielded a black carbonized product. After washing with pyridine (2 × 2 mL), single-crystals could be picked for single-crystal X-ray determination. Crystals can also be obtained by the use of 1.2 mmol (95 mg) pyridine. As the phase is only a side product next to the decomposition of the organic ligands, no suitable elemental analysis and IR spectrum could be achieved.

### 3.2. Crystal Structure Determination

Suitable crystals of **2**, **8**, **9**, **11**, and **12** for single-crystal X-ray analysis were selected out of the solid samples and mixed with viscous, perfluorinated polyalkylether (ABCR). Data collections for the compounds were carried out on a BRUKER AXS Smart Apex 1 diffractometer at 168 K and on a BRUKER AXS Apex II diffractometer at 100 K equipped with graphite monochromators or focusing Helios-mirror optics (Mo-Kα radiation; λ = 0.7107 Å) using the BRUKER AXS Smart Software package or the BRUKER AXS Apex Suite [79]. Further data processing was done with XPREP. All structure solutions were carried out with direct methods using SHELXS [80] and the crystal structures were refined using the program SHELXL [80] together with XSEED [81]. Integrity of symmetry was checked using PLATON. [66] For all compounds, the non-hydrogen atoms were refined anisotropically by least square techniques, all hydrogen atoms were calculated with geometrical constraints regarding their positions.

The crystal structures of${\;}_{\infty }^{2}$[Ln_3_Cl_9_(bipy)_3_], Ln = Pr (**8**), Sm (**9**), show rotational disorder of the two pyridyl-groups of the coordinating bipy molecules along the longitudinal axis, which can be observed by the longitudinal displacement of the thermal ellipsoids in the direction of the rotational disorder. As the spatial distance of the electron-peaks of the disordered pyridyl rings is too small, no stable refinement of the rotational disorder could be introduced. Therefore the disorder will be described by the longitudinal thermal ellipsoids.

The remaining electron density in compound${\;}_{\infty }^{1}$[Ho_2_Cl_6_(bipy)_2_] (**11**) can be explained by a first order phenomenon of the spatial electron density calculations in the FT-electron density map. The six most intense rest electron density peaks radially surround the Ho^3+^ atom in a distance range of 95–105 pm.

For compound${\;}_{\infty }^{2}$[Gd_2_Cl_6_(qpy)_2_(bipy)_2_]·bipy (**12**) rest electron densities of 1 to 2 e × 10^−6^·pm^−3^ can be found in the cavities of the sheet structure. It is assumed that pyridine is statistically spread in the channels of the compound, which cannot be refined. Therefore, the amount of pyridine could not be determined as bulk analytics are not accessible. Similar to compounds **8** and **9**, the crystal structure shows rotational disorder of the two pyridyl-groups of the coordinating bipy molecules along the longitudinal axis, which can be observed by the longitudinal displacement of the thermal ellipsoids in the direction of the rotational disorder. As the spatial distance of the electron-peaks of the disordered pyridyl-rings is too small, no stable refinement of the rotational disorder could be introduced. Therefore the disorder will be described by the longitudinal thermal ellipsoids. Due to crystal quality issues caused by size problems, the R-values are relatively high.

Further information was deposited at the Cambridge Crystallographic Data Centre, CCDC, 12 Union Road, Cambridge CB2 1EZ (Fax: +44-122-333-6033 or E-mail: deposit@ccdc.cam.ac.uk) and the names of the authors and the literature citation may be requested by citing the deposition number CCDC 1001610 (**2**), 1001611 (**8**), 1001612 (**9**), 1001613 (**11**), 1001614 (**12**).

### 3.3. Powder X-ray Diffraction

Compounds **2**, **5**, **8**, **9**, **11**, **12**, **13** and **14** were also investigated on powder samples in sealed Lindemann glass capillaries on a BRUKER AXS D8 Discover powder X-ray diffractometer, equipped with Lynx-Eye detector in transmission geometry. X-ray radiation (Cu-Kα1; λ = 154.06 pm) was focused with a Goebel mirror, Cu-Kα2 radiation was eliminated by the application of a Ni absorber. Diffraction patterns were recorded and analysed using the BRUKER AXS Diffrac-Suite. Furthermore, diffraction data was collected in Debye-Scherrer geometry on a STOE Stadi P powder diffractometer with Ge(111)-monochromatized Cu-Kα radiation (λ = 154.06 pm) using Win-X-Pow software. [82] Traces of LaCl_3_ remnants in (**2**) were identified and refined via Rietveld-method using TOPAS [83] by application of the fundamental parameters approach as reflection profiles (convolution of appropriate source emission profiles with axial instrument contributions as well as crystallite microstructure effects). Temperature dependent diffraction patterns were recorded on a STOE Stadi P powder diffractometer with Ge(111)-mono-chromatized Mo-Kα radiation (λ = 0.7107 Å) and a high-temperature heating device installed on the diffractometer using Win-X-Pow software. Heating rates were 1 °C/min and every 10 °C a diffraction pattern was recorded for 15 min up to 600 °C. The reagents LnCl_3_ were grinded in a mortar in the molecular equivalent of 1/3 LnCl_3_ for Ln = Pr, Sm to bipy and 1/5 for LaCl_3_ to bipy.

### 3.4. Photoluminescence- and Vibrational Spectroscopy

Excitation and emission spectra were recorded with a HORIBA Jobin Yvon Spex Fluorolog 3 spectrometer equipped with a 450 W Xe-lamp, double grated excitation and emission monochromators and a photo multiplier tube (R928P) at RT, using the FluorEssence software. Excitation spectra were corrected for the spectral distribution of the lamp intensity using a photodiode reference detector. Additionally, both excitation and emission spectra were corrected for the spectral response of the monochromators and the detector using correction spectra provided by the manufacturer. The sample was investigated as solid in spectroscopically pure quartz cuvettes in front face mode at room temperature. IR-spectra were recorded with a THERMO Nicolet 380 FT-IR spectrometer in transmission mode using OMNIC 32 software. For that purpose 5 mg of the compounds were mixed with 300 mg of anhydrous KBr and pressed to transparent pellets.

### 3.5. Thermal and Elemental Analysis

Thermal investigations were carried out by simultaneous DTA/TG measurements (Netzsch STA 409, Proteus Software, Selb, Germany), using 15–20 mg of each sample. The compounds were investigated in an inert gas atmosphere (50% Ar and 50% N_2_) and heated from 20 °C to 800 °C with a heating rate of 5 °C·min^−1^ and a constant gas flow of 20 mL·min^−1^ Ar and 20 mL·min^−1^ N_2_.

Carbon, nitrogen and hydrogen elemental analysis were performed using a vario micro cube-system (Elementar Analysensysteme GmbH, Hanau, Germany).

### 3.6. Gas Adsorption Experiments

Volumetric uptake and specific surface areas were determined by nitrogen adsorption-desorption isotherms at −196 °C (N_2_ 99.999%) obtained on a Quantachrome Autosorb 1C instrument. Prior to the adsorption measurements, the samples were outgassed at a vacuum varying between 10^−2^ and 10^−3^ mbar for 12–360 h and at varying temperatures ranging from 100 °C to 315 °C. The thermal treatment was expanded on the BET autosorb instrument to a vacuum of 10^−5^ to 10^−6^ mbar for 4–96 h and for temperatures varying from 70–300 °C.

### 3.7. SEM and EDX Analysis

Morphological investigations were determined on a field emission scanning electron microscope (FE-SEM) ULTRA plus (Zeiss) with a GEMINI e-beam column. Element analysis, mapping and point-ID measurements were carried out using electron dispersive X-ray spectroscopy (EDX) with a silicon drift detector (SDD) X-Max 50 mm^2^ (Oxford Instruments, Oxfordshire, UK) at 15 keV. The instruments were calibrated using calibration files of Oxford Instruments prior to analysis.

## 4. Conclusions

Post-synthetic modification by deliberate thermal treatment beyond activation is shown for the MOF series${\;}_{\infty }^{2}$[Ln_2_Cl_6_(bipy)_3_]·2bipy and${\;}_{\infty }^{3}$[La_2_Cl_6_(bipy)_5_]·4bipy. A combination of elevated temperatures together with different vacuum conditions can be applied to influence the surface morphology of the MOFs. Depending on length and grade of the treatment the intrinsic microporosity of the MOFs is transformed into meso- and macroporosity superseding the microporosity. The surface modification is achieved by control of an evaporation process of molecules incorporated in the pore system of the MOFs that exceeds the release availability of the microporous channels. Increasing pore size is accompanied by a slit-like pore shape. The MOF system${\;}_{\infty }^{2}$[Ln_2_Cl_6_(bipy)_3_] can thereby be used for a morphology control from micro- via meso- to macroporous materials. Deliberate thermal treatment without high vacuum gives the option to activate the MOFs to microporous frameworks without morphology change. Further heating does not just lead to a collapse of the MOF frameworks but offers new highly aggregated dense frameworks depending on the lanthanide ion and temperature. Temperature dependent X-ray powder diffraction shows complicated relationships of the condensed structures. Several new open and dense frameworks were successfully identified for lanthanum, praseodymium and samarium. The MOFs${\;}_{\infty }^{3}$[La_2_Cl_6_(bipy)_5_]·4bipy and${\;}_{\infty }^{2}$[Ln_2_Cl_6_(bipy)_3_]·2bipy thereby prove to be only initial products of the reaction system LnCl_3_/4,4′-bipy. Further heating leads to thermally induced conversion processes that are stepwise condensation reactions by release of the ligand bipy forming LnCl_3_-rich coordination polymers. For La^3+^, the dense framework ^3^_∞_[LaCl_3_(bipy)] (**2**) with sxa-topology is obtained without possible activation to a porous MOF. For Pr^3+^ and Sm^3+^ activation to the porous MOFs${\;}_{\infty }^{2}$[Ln_2_Cl_6_(bipy)_3_], Ln = Pr (**6**), Sm (**7**) is possible prior to a thermal conversion to the dense networks${\;}_{\infty }^{2}$[Ln_3_Cl_9_(bipy)_3_], Ln = Pr (**8**), Sm (**9**). Although identical in constitution to the lanthanum compound **3**, **8** and **9** have different crystal structures. All high-temperature products finally release the remaining equivalents of bipy and return to the crystalline starting reagent LnCl_3_. Therefore, a reaction cycle of LnCl_3_ with bipy to coordination polymers can be performed if the released 4,4′-bipyridine is rapidly evaporated. The identification of additional coordination polymers${\;}_{\infty }^{1}$[Ho_2_Cl_6_(bipy)_2_] (**11**) and${\;}_{\infty }^{2}$[Gd_2_Cl_6_(qtpy)_2_(bipy)_2_]·bipy (**12**) further extends the structural diversity in the reaction system LnCl_3_ and bipy. The latter illustrates a thermal side reaction of the bipy ligand by in-situ formation of qtpy by C-H activation and C-C bond formation of two bipy molecules in *ortho* position. Altogether, the elaboration of thermal treatments of the presented MOF system shows that remarkable surface modifications and thermal reactions can be observed apart from the usual reports on MOF activation and framework collapse.

## Supplementary Materials

Supplementary materials can be accessed at: http://www.mdpi.com/1420-3049/20/07/12125/s1.
