# Supplementary material for: Post-Synthetic Shaping of Porosity and Crystal Structure of Ln-Bipy-MOFs by Thermal Treatment
Source: Molecules. 2015 Jul 3;20(7):12125–53. doi: 10.3390/molecules200712125 (PMC6332306; doi:10.3390/molecules200712125)
Supplement: Supplementary file 1 [file molecules-20-12125-s001.pdf]

Supporting Information

Table S1. Selected interatomic distances (pm) and angles (°) for $^{3}_{\infty}$[LaCl$_3$(bipy)] (**2**).

La(1)-Cl(1)	284.88(4)	Cl(2)-La(1)-Cl(2) ^{II}	71.15(5)
La(1)-Cl(2) $^{\mathrm{I,\,II}}$	284.62(4)	La(1)-Cl(2)-La(1) ^{II}	108.85(13)
La(1)-Cl(2) ^{III}	292.41(7)	Cl(1)-La(1)-Cl(1) ^{I}	78.10(14)
La(1)-N(1)	278.4(2)	Cl(2)-La(1)-Cl(2) ^{III}	80.30(5)
C(3)-C(3) ^{V}	149.2(5)	Cl(1)-La(1)-Cl(1) ^{II}	77.41(13)
(C=C,C=N, range)	134.7(3)–140.2(3)	N(1)-La(1)-Cl(2) ^{II}	69.45(5)
La(1)-Cl(1)-La(1) ^{IV}	180.00	N(1)-La(1)-Cl(2) ^{I}	74.02(5)
N(1)-Cl(1)-N(1) ^{I}	139.70(13)		

Symmetry operations: ^{I} 1-x, y, ½-z; ^{II} 1-x, 1-y, −z; ^{III} x, 1-y, ½+z, ^{IV} 1-x, 2-y, −z, ^{V} 3/2-x, 2-y, z.

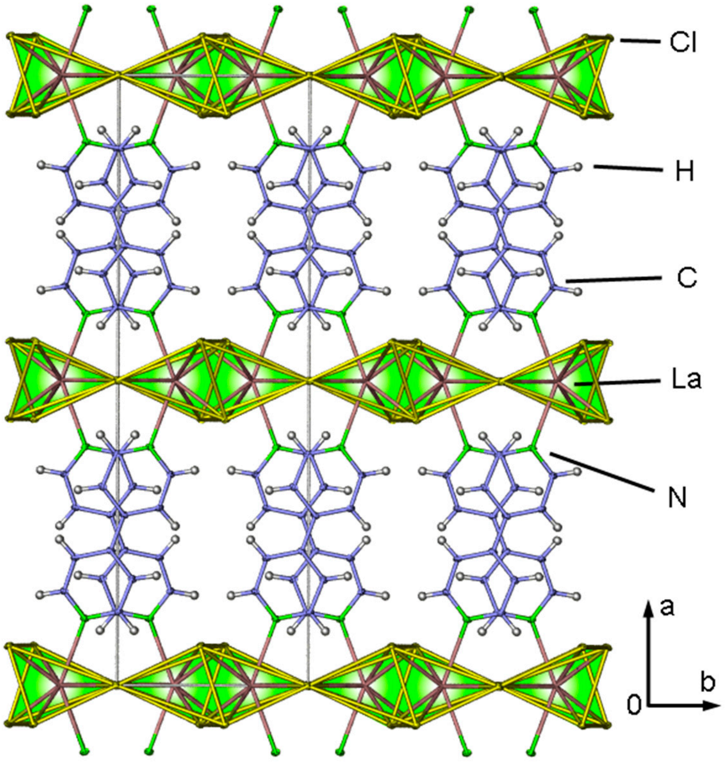

Figure S1. Depiction of the single-crystal structure of $^{3}_{\infty}$[LaCl$_3$(bipy)] (**2**) with view along c-axis.

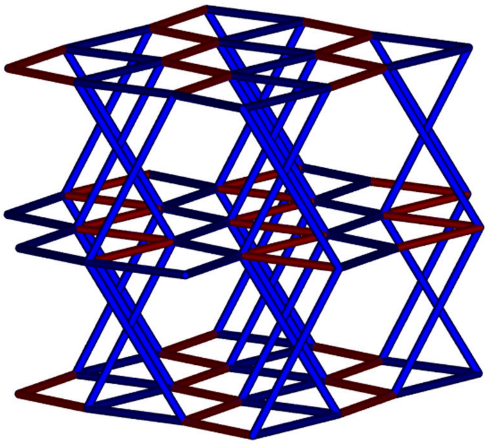

Figure S2. Depiction of the sxa network topology of $^{3}_{\infty}$[LaCl$_3$(bipy)] (**2**).

Table S2. Selected interatomic distances (pm) and angles (°) for $^{2}_{\infty}[Ln_3Cl_9(bipy)_3]$, Ln = Pr (**8**), Sm (**9**).

	**Pr (8)**	**Sm (9)**
Ln1-Cl1	296.4(2)	293.22(10)
Ln1-Cl2	278.1(2)	274.58(14)
Ln1-Cl3	276.9(3)	273.1(2)
Ln1-Cl4	279.8(3)	276.5(2)
Ln1-Cl4^{II}	285.7(3)	281.9(2)
Ln2-Cl1	296.6(2)	292.54(14)
Ln2-Cl3	291.9(3)	289.6(2)
Ln2-Cl5	269.4(3)	266.5(2)
Ln1-N1	257.4(7)	253.2(4)
Ln2-N2	261.7(9)	256.0(5)
C3-C3 ^{I}	149(2)	149.7(9)
C8-C8 ^{II}	149(2)	149.3(12)
C-(C=C,C=N, range)	130.9(11)–140(2)	131.1(6)–138.5(7)
N1-Ln1-N1	157.4(4)	157.3(3)
N2-Ln2-N2	153.6(4)	153.6(3)
Ln1-N1-C3	167.74(13)	167.10(10)
Ln2-N2-C8	170.68(13)	171.08(10)
Ln1-Cl3-Ln2	99.39(9)	99.16(5)
Ln1-Cl2-Ln2	99.67(11)	99.97(7)
Ln1-Cl1-Ln2	94.06(5)	94.06(3)
Ln1-Cl1-Ln1	91.60(6)	91.64(4)
Ln1-Cl4-Ln1	107.91(8)	108.22(5)

Symmetry operations: ^{I} x, −y, 2-z; ^{II} x, −y, z-1/2.

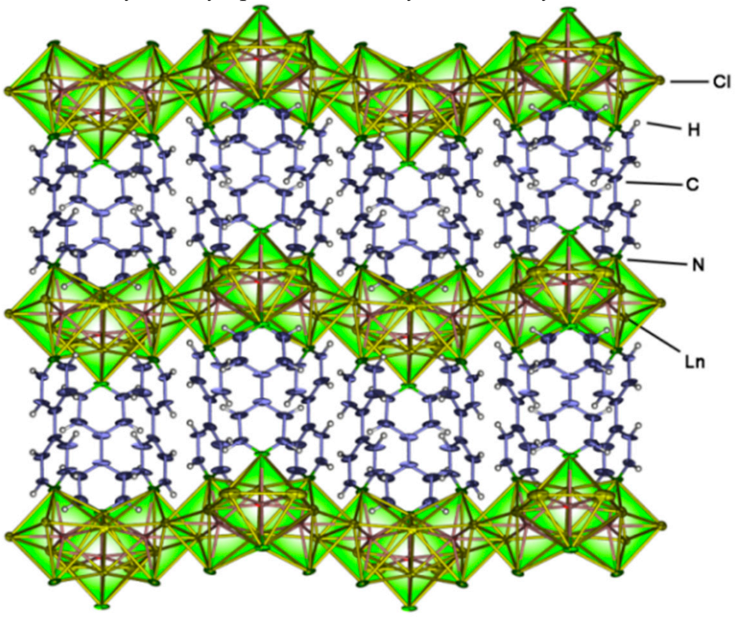

Figure S3. Depiction of a two-dimensional sheet of the crystal structure of $^{2}_{\infty}[Ln_2Cl_6(bipy)_2]$ with Ln = Pr (**6**), Sm (**7**). Thermal ellipsoids are depicted with 50% probability.

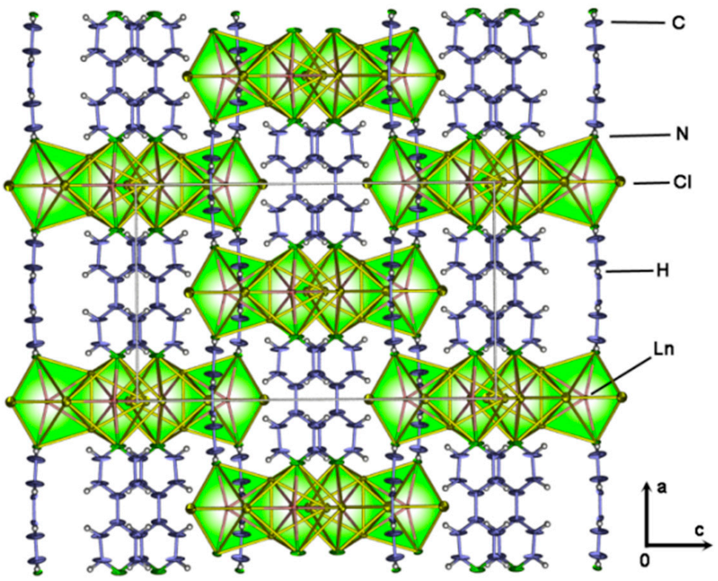

Figure S4. View along the b-axis of the crystal structure of $^{2}_{\infty}[Ln_3Cl_9(bipy)_3]$ for Ln = Pr (**8**), Sm (**9**).

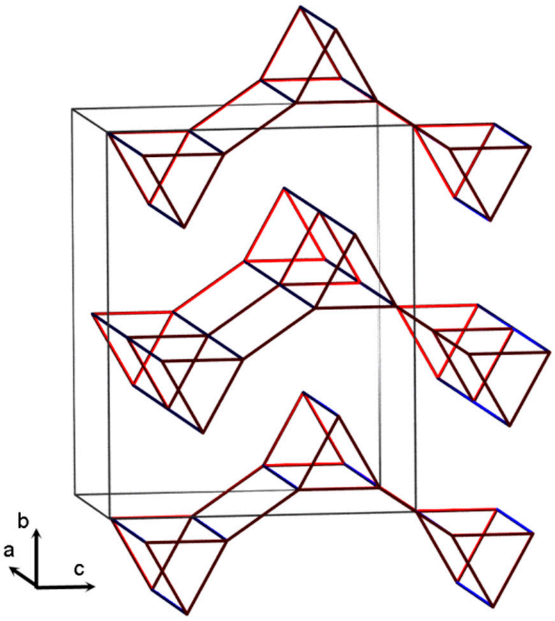

Figure S5. Depiction of the topology of the sheet structure of $^{2}_{\infty}[Ln_3Cl_9(bipy)_3]$ with Pr (**8**) and Sm (**9**). Ln-Cl double bridges are marked in red, Ln-bipy bridges are marked in blue.

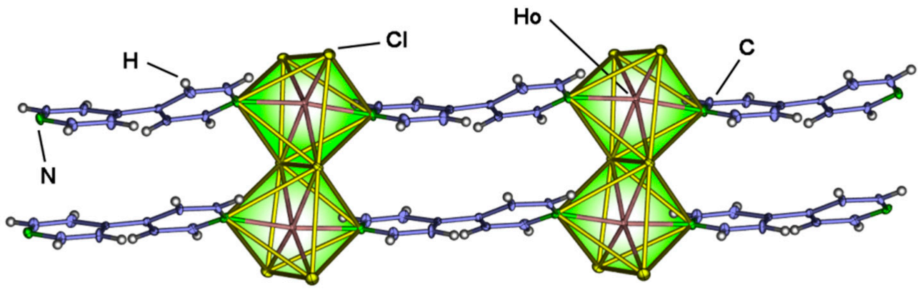

Figure S6. Depiction of the 4,4′-bipyridine connected octahedral, dimeric units $Ho_2Cl_6N_2$ of the double-chain structure of $^{1}_{\infty}[Ho_2Cl_6(bipy)_2]$ (**11**).

Table S3. Interatomic distances (pm) and angles (°) for $^{1}_{\infty}[Ho_2Cl_6(bipy)_2]$ (**11**).

Ho1-Cl1	273.6 (2)	Cl2-Ho1-Cl1	79.09(4)
Ho1-Cl2	270.1(2)	Cl3-Ho1-Cl1	90.19(5)
Ho1-Cl3	252.1(2)	Cl3-Ho1-Cl2	168.89(5)
Ho1-Cl4	250.3(2)	Cl4-Ho1-Cl1	167.42(5)
Ho1-N1	244.0(5)	Cl4-Ho1-Cl2	88.36(5)
Ho1-N2	243.5(5)	Cl4-Ho1-Cl3	102.30(5)
Ho2-Cl1	270.5(2)	N2-Ho1-N1	172.7(2)
Ho2-Cl2	269.5(2)	Cl2-Ho2-Cl1	79.74(4)
Ho2-Cl5	252.1(2)	Cl5-Ho2-Cl1	92.16(5)
Ho2-Cl6	254.0(2)	Cl5-Ho2-Cl2	171.89(5)
Ho2-N3	244.6(5)	Cl6-Ho2-Cl1	164.28(5)
Ho2-N4	245.7(5)	Cl6-Ho2-Cl2	84.62(5)
C3-C6	149.3(8)	Cl6-Ho2-Cl5	103.49(5)
C13-C16	147.4(8)	N4-Ho2-N3	172.28(16)
C=C, C =N range	132.8(8)-140.7(8)	Ho2-Cl1-Ho1	100.01(4)
		Ho2-Cl2-Ho1	101.15(5)

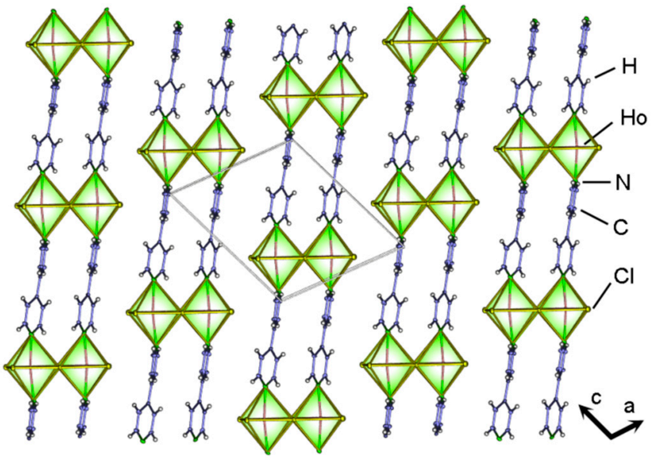

Figure S7. Depiction of a single sheet of the *ac*-plane of $^{1}_{\infty}[Ho_2Cl_6(bipy)_2]$ (**11**).

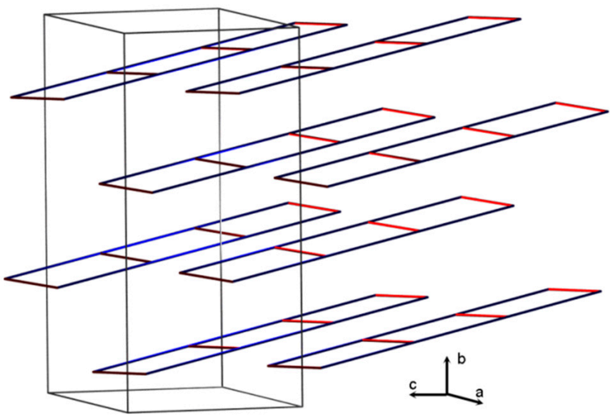

Figure S8. Depiction of the topology of the double strand structure of $^{1}_{\infty}[Ho_2Cl_6(bipy)_2]$ (**11**). Ho-Cl double bridges are marked in red. Ho-bipy bridges are marked in blue.

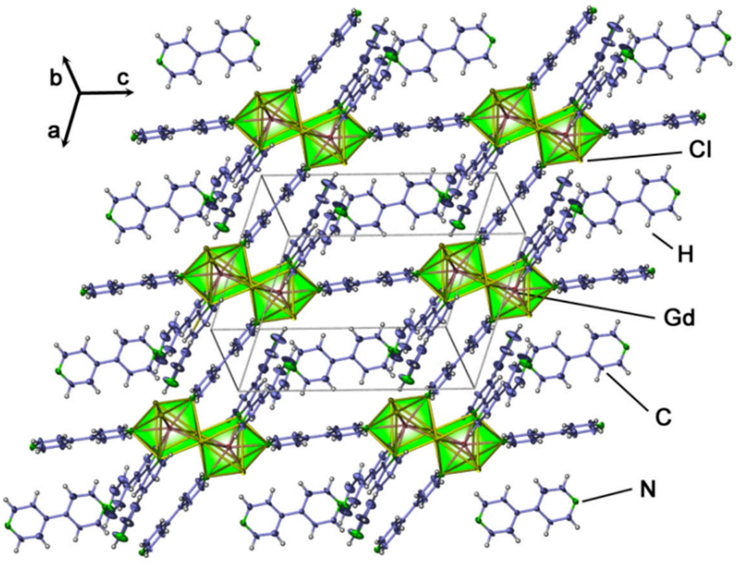

Figure S9. Depiction of the two-dimensional sheet structure of $^{2}_{\infty}[Gd_2Cl_6(qtpy)_2(bipy)_2]\cdot bipy$ (**12**). Thermal ellipsoids are depicted with 30% probability.

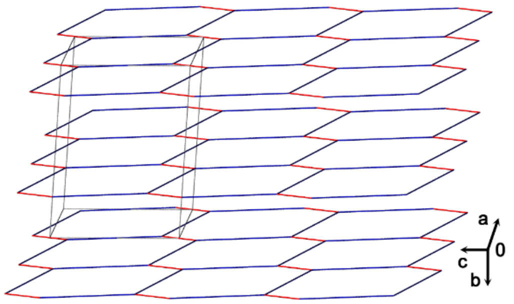

Figure S10. Depiction of the $\{6^3\}$-hcb topology of $^{2}_{\infty}[Gd_2Cl_6(qtpy)_2(bipy)_2]\cdot bipy$ (**12**). Gd-Cl double bridges are marked in red. Gd-bipy bridges are marked in blue.

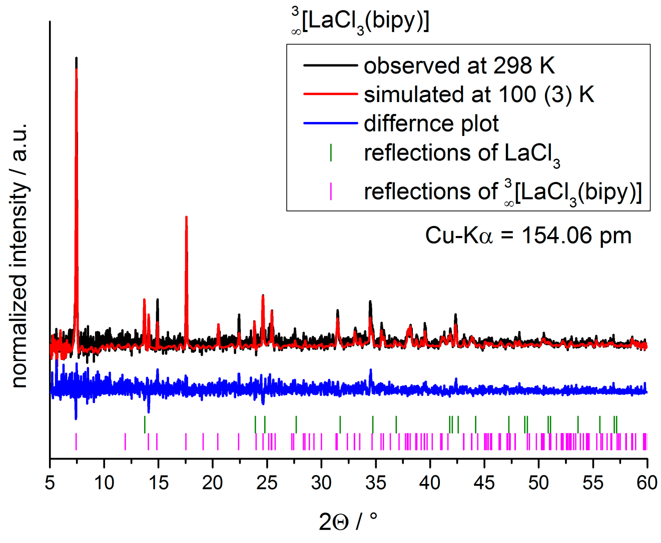

Figure S11. Plot of Rietveld refinement of $^{3}_{\infty}[LaCl_3(bipy)]$ (**2**). Observed diffraction pattern (black), simulated diffraction pattern (red), difference plot (blue), reflection positions of $LaCl_3$ (green) and $^{3}_{\infty}[LaCl_3(bipy)]$ (**2**) (magenta).

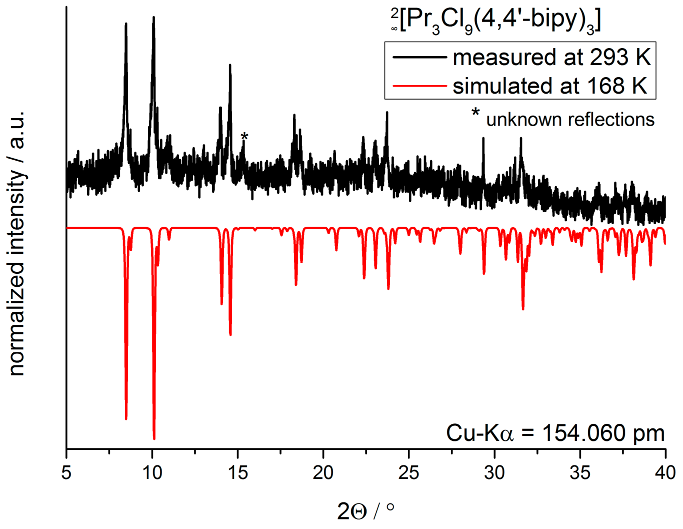

Figure S12. Plot of the observed diffraction pattern of compound $^2_\infty$[Pr_3Cl_9(bipy)$_3$] (**8**) compared to a simulated diffraction pattern from single-crystal data of **8**.

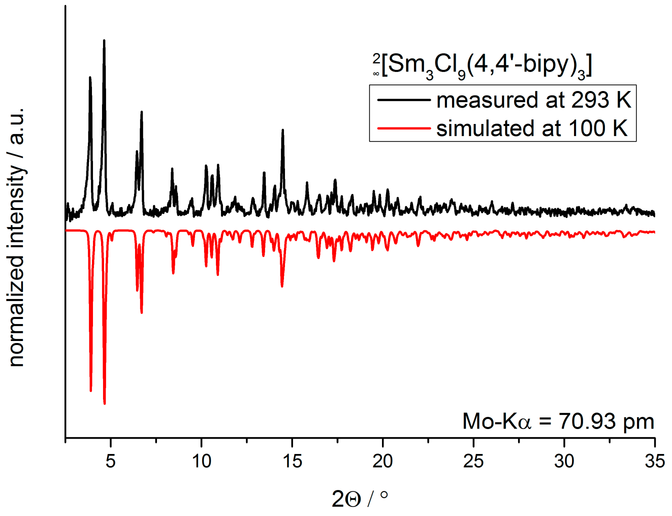

Figure S13. Plot of the observed diffraction pattern of $^2_\infty$[Sm_3Cl_9(bipy)$_3$] (**9**) compared to a simulated diffraction pattern from single.

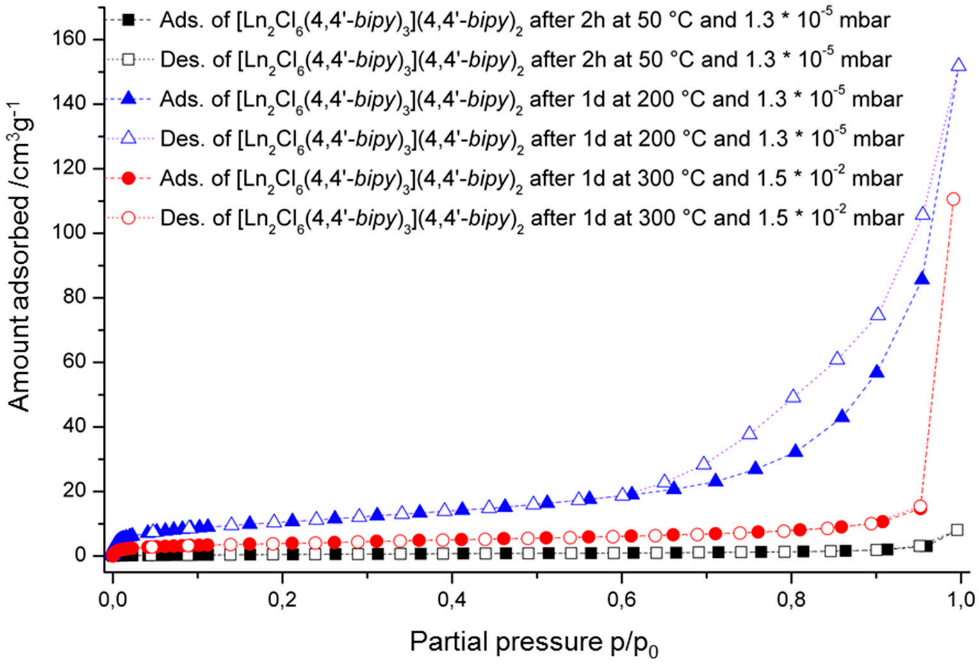

Figure S14. Adsorption isotherms of the MOF $^{2}_{\infty}$[Eu_2Cl_6(bipy)$_3$]·2bipy (**13**) for different activation canditions. High vacuum proves the key to the formation of mesopores (200 °C for 24 h at 10^{-5} mbar) for N_2 adsorption (77 K), whereas higher temperatures of 300 °C at low vacuum prove insufficient.

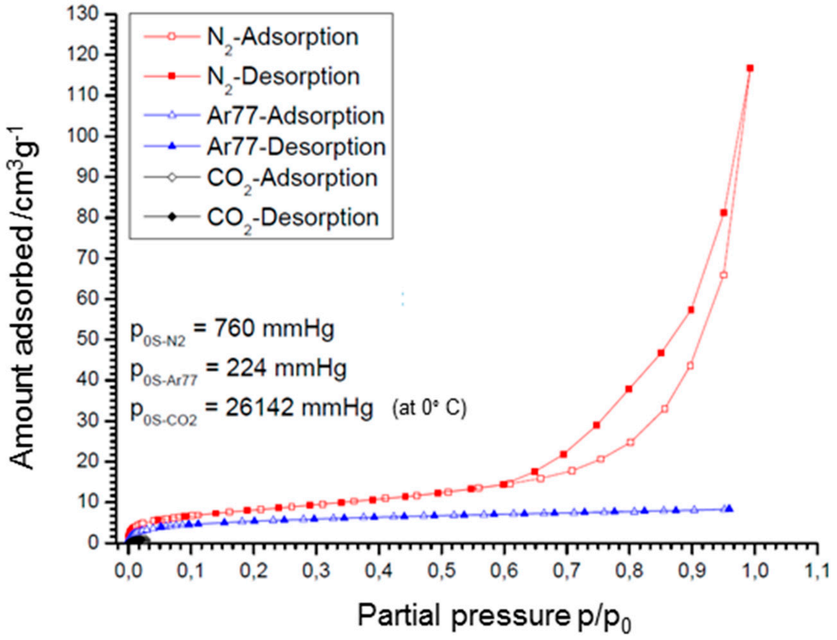

Figure S15. Adsorption isotherms of the mixed-metal MOF $^{2}_{\infty}$[$Eu_{0.8}Tb_{1.2}Cl_6$(bipy)$_3$]·2bipy (**14**) for the different gases subsequent to activation at 200 °C for 18 h at 10^{-6} mbar; N_2 adsorption indicates the formation of mesopores.

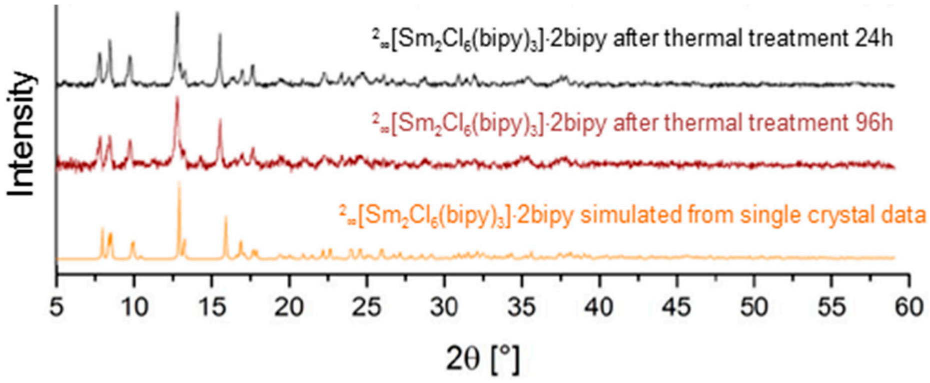

Figure S16. X-ray powder diffractograms of $^{2}_{\infty}[Sm_2Cl_6(bipy)_3]\cdot 2bipy$ (**5**) subsequent to the surface modification at 300 °C for 24 h at 10^{-6} mbar (top), after 96 h (mid) and a simulated pattern from single crystal data (bottom) indicating retaining of the network structure.
